# The singular *Corynebacterium glutamicum* Emb arabinofuranosyltransferase polymerises the α(1 → 5) arabinan backbone in the early stages of cell wall arabinan biosynthesis

**DOI:** 10.1016/j.tcsw.2018.06.003

**Published:** 2018-06-20

**Authors:** Monika Jankute, Luke J. Alderwick, Alice R. Moorey, Maju Joe, Sudagar S. Gurcha, Lothar Eggeling, Todd L. Lowary, Anne Dell, Poh-Choo Pang, Tiandi Yang, Stuart Haslam, Gurdyal S. Besra

**Affiliations:** a Institute of Microbiology and Infection, School of Biosciences, University of Birmingham, Edgbaston, Birmingham B15 2TT, UK; b Institute of Bio- and Geosciences, IBG-1: Biotechnology, Forschungszentrum Jülich GmbH, Jülich D-52425, Germany; c Department of Chemistry, Centennial Centre for Interdisciplinary Science, University of Alberta, Alberta T6G 2G2, Canada; d Department of Life Sciences, Imperial College London, London SW7 2AZ, UK

**Keywords:** Arabinogalactan, Cell envelope, Corynebacteria, Mycobacteria, Glycosyltransferase

## Abstract

The arabinan-containing polysaccharides, arabinogalactan (AG) and lipoarabinomannan (LAM), are key cell wall components of the *Corynebacterineae*, which include Corynebacteria, Norcadia and Mycobacteria. Both AG and LAM contain elaborate arabinan domains composed of distinct structural motifs. Mycobacterial EmbA, EmbB and EmbC, collectively known as the Emb proteins, have been identified as arabinosyltransferases (Ara*f*Ts), which are targeted by the front-line anti-tubercular drug ethambutol. Previous studies have established that EmbA and EmbB play a role in the synthesis of the characteristic terminal hexa-arabinosuranosyl motif, whilst EmbC is involved exclusively in the biosynthesis of LAM. Herein, we have investigated the role of the singular Emb protein from *Corynebacterium glutamicum* through the detailed biochemical and chemical analysis of a double *ΔaftAΔemb* mutant, where the priming Cg-AftA protein, which generates the substrate for Cg-Emb has been deleted. Analysis of its cell wall revealed a complete absence of arabinose resulting in a truncated cell wall containing only a galactan backbone accompanied with complete loss of cell wall bound mycolates. *In vitro* cell-free assays using *C. glutamicumΔaftA, C. glutamicumΔemb, C. glutamicumΔaftAΔemb* and *C. glutamicumΔaftBΔaftD* and two synthetic acceptors, which mimick the arabinofuranose (Ara*f*) “primed” galactan chain, demonstrated that Cg-Emb is able to transfer an Ara*f* residue to the C5 of the Ara*f* positioned on the synthetic acceptor(s). These results indicate that Cg-Emb acts as an α(1 → 5) Ara*f*T and elongates the arabinan core during the early stages of arabinan biosynthesis in *C. glutamicum*.

## Introduction

Tuberculosis (TB) is an infection of global significance which was responsible for over 1.3 million deaths in 2016 alone (World Health Organization, 2017). The causative pathogen, *Mycobacterium tuberculosis*, results in the highest number of fatalities from a single infectious agent (World Health Organization, 2017). Whilst, TB treatments have been available since the 1960s, the prevalence of the disease remains high. The long duration and unpleasant side effects of treatment regimens result in poor patient compliance, which in part may have contributed to the rise of multi-drug resistant (MDR) and extensively drug resistant (XDR) strains of *M. tuberculosis*. In order to address the global demand for effective TB treatments new chemotherapeutic targets are actively being explored (Boot et al., 2018). The hallmark of mycobacterial species is their impermeable, lipid-rich cell envelope that contributes to their resilience and intrinsic resistance to a range of common antibiotics. As a result, the synthesis of the intricate cell wall matrix provides excellent drug targets for some of the widely used TB drugs, including ethambutol (EMB) and isoniazid (Banerjee et al., 1994, Belanger et al., 1996, Telenti et al., 1997). The mycobacterial cell wall consists of three main components that together form the mycolyl-arabinogalactan-peptidoglycan (mAGP) complex: a mycolic acid layer formed of long chain fatty acids, a highly branched arabinogalactan (AG) and a peptidoglycan (PG) backbone. This macromolecular structure is further interspersed with glycolipids, such as lipomannan (LM) and lipoarabinomannan (LAM), both of which have crucial structural and immunomodulatory roles (Mishra et al., 2011).

The AG macromolecule is an integral component of the mycobacterial cell wall, forming the central portion of the mAGP. Bound to MurNGlyc residues by a linker unit, α-1-Rhap-(1 → 3)-α-D-GlcNAc-(1 → P), AG is structurally important as it connects the mycolic acid layer to the PG backbone (McNeil et al., 1990). Galactofuranosyltransferases (Gal*f*T), Gl*f*T1 and Gl*f*T2 are responsible for the polymerisation of galactofuranose (Gal*f*) residues to the PG linker unit, with Gl*f*T2 elongating a linear chain of approximately 30 Gal*f* residues in an alternate β(1 → 5) and β (1 → 6) fashion (Alderwick et al., 2008, Kremer et al., 2001, Rose et al., 2006). Synthesis of the galactofuranose chain is followed by the addition of three arabinofuranose (Ara*f*) residues to the 8th, 10th and 12th Gal*f* residues (Alderwick et al., 2005). The first Ara*f* residues are transferred from the arabinosyl donor decaprenylphosphoryl-D-arabinose (DPA) by the *Corynebacterium glutamicum* arabinofuranosyltransferase (Ara*f*T) AftA to C-5 of the β (1 → 6) Gal*f* residues (Alderwick et al., 2006b, Lee et al., 1995). Elongation through the addition of further α(1 → 5) linked Ara*f* residues from DPA occurs to form a linear arabinan backbone. The arabinan chain is then bifurcated at approximately the 13th Ara*f* residue by *Mycobacterium smegmatis* AftC with the introduction of an α(1 → 3) arabinosyl linkage (Birch et al., 2008). Over-expression experiments suggested that AftD also acts as a branching α(1 → 3) Ara*f*T in *M. smegmatis* (Skovierová et al., 2009). However, recent studies in *C. glutamicum* clearly demonstrate that AftD is an α(1 → 5) Ara*f*T, which further elongates the bifurcation strands of arabinan in AG (Alderwick et al., 2018a)*.* The non-reducing terminus of the AG arabinan is then completed by Cg-AftB with β (1 → 2) activity, that caps the terminal arabinan domain residues (Seidel et al., 2007). This results in a characteristic hexaarabinofuranosyl (Ara_6_) motif that subsequently serves as the site of mycolic acid esterification (McNeil et al., 1991).

The Emb Ara*f*Ts – EmbA, EmbB and EmbC – are the known targets of the front-line anti-TB drug ethambutol (Belanger et al., 1996, Telenti et al., 1997). Deletion studies have confirmed that impairment of different *emb* genes results in different effects on cell wall biosynthesis in *M. smegmatis*. Specifically, disruption of both *embA* and *embB* affected the terminal-Ara_6_ motif that serves as a template for subsequent mycolylation (Escuyer et al., 2001). Both EmbA and EmbB are predicted to act as α(1 → 3) Ara*f*Ts, but no direct evidence for this has been uncovered (Bhamidi et al., 2008). Whilst, EmbA and EmbB play a role in AG biosynthesis, disruption of *embC* results in reduced synthesis of LAM, and thus EmbC is suggested to add Ara*f* residues exclusively to the arabinan domain of LAM (Shi et al., 2006). Interestingly, *C. glutamicum*, possesses only one *emb* gene that is non-essential and is involved in arabinan synthesis, the deletion of which results in a viable but slow-growing mutant with a highly truncated AG-glycan possessing single arabinose residues attached to the galactan backbone (Alderwick et al., 2005). The ability to create arabinan-deficient strains of *C. glutamicum* makes it an ideal candidate for deletion studies and research into AG biosynthesis (Alderwick et al., 2006b, Alderwick et al., 2006a).

In the present study, we have investigated the biosynthesis of AG in *C. glutamicum* by generating a double deletion mutant of *aftA* and *emb*, which through biochemical and chemical analyses has revealed that Cg-Emb acts as an α(1 → 5) Ara*f*T and transfers Ara*f* residues from DPA to the Ara*f*-primed galactan chain in *C. glutamicum*.

## Materials and methods

### Synthesis of MJ-13-77 and MJ-14-01

All reagents were purchased from commercial sources without further purification, while reaction solvents were purified using a PURESOLV-400 system (Innovative Technology Inc., Newburyport, MA). All reactions were carried out in oven-dried glassware under a positive pressure of argon and monitored by TLC Silica Gel 60 F_254_ (0.25 mm, E. Merck) unless otherwise indicated. Plates were visualized under UV light and/or stained with a solution of *p*-anisaldehyde or 5% H_2_SO_4_ in ethanol. Column chromatography was performed using Silicycle UltraPure silica gel (SiliaFlash® P60, 40–63 μm, Cat# R12030 B). The ratio between silica gel and crude product ranged from 100:1 to 20:1 (w/w). Optical rotations were measured in a microcell (10 cm, 1 mL) at 22 ± 2 °C and are in units of degree·mL/(g·dm). Organic solutions were concentrated under vacuum at temperature below 50 °C on a rotary evaporator. ^1^H NMR spectra were recorded at 500 MHz, and chemical shifts were referenced to CDCl_3_ (7.26 ppm), or D_2_O (4.78 ppm). ^1^H NMR data are reported as though they are first order and the peak assignments were made on the basis of 2D-NMR (^1^H–^1^H COSY and HMQC) experiments. ^13^C NMR spectra were recorded at 125 MHz, and ^13^C chemical shifts are referenced to CDCl_3_ (77.23) or external acetone (31.07, D_2_O). Electrospray mass spectra were recorded on samples suspended in mixtures of THF with CH_3_OH and added NaCl.

#### Octyl 2,3-di-*O*-benzoyl*-*β-D-galactofuranoside (4)

To a solution of compound 3 (Completo and Lowary, 2008) (1.34 g, 4.6 mmol) in acetone (40 mL) and 2,2-dimethoxypropane (20 mL) at room temperature was added *p*-TSA·H_2_O (13 mg, 0.07 mmol) and the solution was stirred for 90 min. The reaction was then quenched by adding four drops of triethylamine and the mixture was then concentrated on a rotary evaporator. The syrupy residue was then dissolved in pyridine (10 mL) and cooled to 0 °C followed by the addition of benzoyl chloride (1.3 mL, 11.2 mmol) dropwise. The reaction mixture was allowed to warm to room temperature and stirred overnight. The reaction was quenched by the addition of chilled water (50 mL) and extracted with CH_2_Cl_2_ (60 mL). The CH_2_Cl_2_ layer was washed with 10% aqueous copper sulfate solution (30 mL × 4), water (40 mL). The separated CH_2_Cl_2_ layer was dried (Na_2_SO_4_), and concentrated. The syrupy residue obtained was dissolved in a solution of acetic acid: water: THF (3:1.5:1.5; 50 mL) and heated at 55–60 °C for 9 h. The reaction mixture was then directly concentrated on rotary evaporator at 50 °C and the residue was purified by column chromatography (6.5:3.5 hexanes–EtOAc) to yield 4 (1.4 g, 61% over three steps) as a thick syrup. [α]_D_ +9.4 (*c* = 0.9, CHCl_3_); *R_f_* 0.22 (6.5:3.5 hexanes–EtOAc); ^1^H NMR (500 MHz, CDCl_3_, δ_H_) 8.12–8.02 (m, 4H), 7.61–7.56 (m, 2H), 7.50–7.42 (m, 4H), 5.58 (dd, *J* = 4.6, 1.2 Hz, 1H, H-3), 5.50 (d, *J* = 1.2 Hz, 1H, H-2), 5.25 (s, 1H, H-1), 4.32 (dd, *J* = 4.5, 3.4 Hz, 1H, H-4), 4.17–4.12 (m, 1H, H-5), 3.90–3.70 (m, 3H, H-6, 6′, OCH_2_), 3.54 (ddd, *J* = 9.5, 6.3, 6.3 Hz, 1H, OCH_2_), 2.50 (br.s, 2H), 1.70–1.58 (m, 2H), 1.42–1.20 (m, 10H), 0.86 (dd, *J* = 6.9 Hz, 3H); ^13^C NMR (125 MHz, CDCl_3_, δ_C_) 166.1, 165.3, 133.6, 129.9(3), 129.9, 129.1(4), 129.1(0), 128.5(4), 128.5, 105.7, 84.2, 81.3, 78.0, 70.8, 67.7, 64.4, 31.8, 29.6, 29.4, 29.3, 26.2, 22.6, 14.1. HRMS (ESI) *m/z* calcd for (M + Na) C_28_H_36_O_8_Na: 523.2302. Found: 523.2304.

#### Octyl 2,3-di-O-benzoyl-6-O-t-butyldiphenylsilyl-β-D-galactofuranoside (5)

To a solution of 4 (1.37 g, 2.5 mmol) in pyridine (20 mL) and CH_2_Cl_2_ (10 mL) at 0 °C was added *t*-butyldiphenylsilyl chloride (0.97 mL, 3.8 mmol) dropwise. The solution was then stirred overnight with warming to room temperature before CH_3_OH (0.4 mL) was added. After stirring for 30 min, the solution was poured into a saturated aqueous NaHCO_3_ (25 mL) and then extracted with CH_2_Cl_2_ (50 mL). The organic layer was washed with brine, dried (Na_2_SO_4_), filtered and concentrated to a residue that was purified by chromatography (87:13 hexanes–EtOAc) to yield 5 (1.9 g, 96%) as a thick syrup. [α]_D_ −1.4 (*c* = 0.7, CHCl_3_); *R_f_* 0.51 (4:1 hexanes–EtOAc); ^1^H NMR (500 MHz, CDCl_3_, δ_H_) 8.12–8.02 (m, 4H), 7.70–7.63 (m, 4H), 7.63–7.54 (m, 2H), 7.48–7.31 (m, 10H), 5.62 (d, *J* = 1.3 Hz, 1H, H-3), 5.48 (d, *J* = 1.4 Hz, 1H, H-2), 5.24 (s, 1H, H-1), 4.50 (dd, *J* = 5.0, 2.6 Hz, 1H, H-4), 4.19–4.14 (m, 1H, H-5), 3.88–3.72 (m, 3H, H-6, 6′, OCH_2_), 3.50 (ddd, *J* = 9.4, 6.3, 6.3 Hz, 1H, OCH_2_), 2.45 (br.s, 1H), 1.68–1.56 (m, 2H), 1.43–1.20 (m, 10H), 1.04 (s, 9H), 0.86 (dd, *J* = 6.9 Hz, 3H); ^13^C NMR (125 MHz, CDCl_3_, δ_C_) 165.8, 165.4, 135.5, 133.4(1), 133.4, 133.2(2), 133.2, 129.9(2), 129.9, 129.7(4), 129.7(1), 129.4, 129.3, 128.5, 128.4, 127.7(2), 127.7(0), 105.6, 82.1, 81.7, 78.1, 70.9, 67.5, 64.8, 31.8, 29.6, 29.4, 29.3, 26.8, 26.2, 22.6, 19.2, 14.1. HRMS (ESI) *m/z* calcd for (M + Na) C_44_H_54_O_8_SiNa: 761.3480. Found: 761.3482.

#### Octyl 2,3,5-tri-*O*-benzoyl-α-D-D-arabinofuranosyl-(1 → 5)-2,3-di-*O*-benzoyl-β-D-galactofuranoside (8)

Thioglycoside 6 (Joe et al., 2011) (0.35 g, 0.62 mmol) and alcohol 5 (0.4 g, 0.51 mmol) were dried over phosphorus pentoxide under vacuum for 6 h and then dissolved in CH_2_Cl_2_ (20 mL) and the resulting solution was cooled to 0 °C. Powdered 4 Å molecular sieves (0.25 g) were added and the suspension was stirred for 30 min at 0 °C before *N*-iodosuccinimide (0.14 g, 0.62 mmol) and silver triflate (16 mg, 0.06 mmol) were added. The reaction mixture was stirred for 25 min, neutralized with Et_3_N, diluted with CH_2_Cl_2_ (15 mL) and filtered through Celite. The filtrate was washed successively with a saturated aqueous Na_2_S_2_O_3_ (25 mL) and water before being dried (Na_2_SO_4_), filtered and concentrated. The crude residue (crude 7) was dissolved in a solution of pyridine–THF (4:1, 15 mL) at 0 °C and 70% HF–pyridine (0.6 mL) was added dropwise. The reaction mixture was stirred for 36 h while warming to room temperature before being diluted with EtOAc, poured into a saturated aqueous NaHCO_3_ (25 mL) and extracted with EtOAc (40 mL). The organic layer was washed with water, dried (Na_2_SO_4_), filtered and concentrated to give crude syrup that was purified by column chromatography (4:1, hexanes–EtOAc) to afford 8 (0.37 g, 76% over 2 steps) as a white foam. [α]_D_ +1.9 (*c* = 0.32, CHCl_3_); *R_f_* 0.16 (4:1 hexanes–EtOAc); ^1^H NMR (500 MHz, CDCl_3_, δ_H_) 8.10–7.94 (m, 10H), 7.60–7.30 (m, 15H), 5.77 (s, 1H), 5.71 (dd, *J* = 5.5, 1.6 Hz, 1H), 5.70–5.61 (m, 2H), 5.49 (d, *J* = 1.5 Hz, 1H), 5.26 (s, 1H), 4.80–4.76 (m, 2H), 4.65 (dd, *J* = 12.7, 6.5 Hz, 1H), 4.42 (dd, *J* = 5.5, 4.1 Hz, 1H), 4.33 (ddd, *J* = 7.3, 3.9, 3.9 Hz, 1H), 4.00–3.94 (m, 1H), 3.92–3.86 (m, 1H), 3.76 (ddd, *J* = 9.6, 6.8, 6.8 Hz, 1H), 3.52 (ddd, *J* = 9.5, 6.4, 6.4 Hz, 1H), 2.92 (dd, *J* = 9.2, 3.9 Hz, 1H), 1.70–1.60 (m, 2H), 1.43–1.22 (m, 10H), 0.86 (dd, *J* = 6.9 Hz, 3H); ^13^C NMR (125 MHz, CDCl_3_, δ_C_) 166.2, 165.8, 165.7, 165.6, 165.2, 133.5, 133.4, 133.3, 133.1, 129.9, 129.8, 129.7(8), 129.6, 129.2(2), 129.2(0), 129.0(1), 129.0, 128.4(4), 128.4(2), 128.4(0), 128.4, 107.2, 105.6, 83.0, 82.2, 82.0, 81.1, 79.4, 77.7, 77.5, 67.7, 63.9, 63.6, 31.8, 29.5, 29.4, 29.3, 26.2, 22.7, 14.1. HRMS (ESI) *m/z* calcd for (M + Na) C_54_H_56_O_15_Na: 967.3511. Found: 967.3509.

#### *p*-Thiotolyl 2,3-di-*O*-benzoyl-5-*O*-levulinoyl-6-*O*-*t*-butyldiphenylsilyl-β-D-galactofuranoside (9)

A mixture of 17 (1.3 g, 1.6 mmol), levulinic acid (0.27 mL, 2.6 mmol), 1,3-dicyclohexylcarbodiimide (0.54 g, 2.6 mmol), and (4-dimethylamino)pyridine (0.11 g, 0.9 mmol) in CH_2_Cl_2_ (38 mL) was stirred for 1 h. The reaction mixture was diluted with CH_2_Cl_2_ (5 mL), filtered through Celite, washed with a saturated aqueous NaHCO_3_ (15 mL), and brine (15 mL). The organic layer was then dried (Na_2_SO_4_), filtered, and concentrated to give a residue, which was purified by column chromatography (4:1, hexanes–EtOAc) to afford 9 (1.42 g, 98%) as a thick syrup. [α]_D_ −32.6 (*c* = 0.65, CHCl_3_); *R_f_* 0.24 (4:1 hexanes–EtOAc); ^1^H NMR (500 MHz, CDCl_3_, δ_H_) 8.15–8.04 (m, 4H), 7.70–7.50 (m, 6H), 7.50–7.36 (m, 8H), 7.36–7.30 (m, 4H), 7.10–7.05 (m, 2H), 5.68 (d, *J* = 2.0 Hz, 1H, H-1), 5.64 (dd, *J* = 2.0, 2.0 Hz, 1H, H-2), 5.57–5.46 (m, 2H, H-3, H-5), 4.91 (dd, *J* = 4.7, 4.7 Hz, 1H, H-4), 3.95–3.85 (m, 2H, H-6, 6′), 2.68–2.63 (m, 2H), 2.58–2.52 (m, 2H), 2.32 (s, 3H), 2.09 (s, 3H), 1.01 (s, 9H); ^13^C NMR (125 MHz, CDCl_3_, δ_C_) 206.0, 171.9, 165.3(5), 165.3, 138.0, 135.6, 135.5, 133.5, 133.0(6), 133.0(1), 130.0, 129.9, 129.7, 129.6, 129.1(4), 129.1(0), 128.5, 127.7, 91.2, 82.1, 80.4, 77.5, 72.4, 62.1, 38.0, 29.7, 28.0, 26.8, 26.7, 21.1, 19.2. HRMS (ESI) *m/z* calcd for (M + Na) C_48_H_50_O_9_SiSNa: 853.2837. Found: 853.2837.

#### Octyl 2,3-di-*O*-benzoyl-5-*O*-levulinoyl-6*-O-t-*butyldiphenylsilyl*-*β-D-galactofuranosyl-(1 → 6)-2,3-di-*O*-benzoyl-[5-*O*-(2,3,5-tri-*O*-benzoyl*-α*-D-arabinofuranosyl-)]-β-D-galactofuranoside (10)

Thioglycoside 9 (0.23 g, 0.28 mmol) and alcohol 8 (0.22 g, 0.23 mmol) were dried over phosphorus pentoxide under vacuum for 6 h and then dissolved in CH_2_Cl_2_ (10 mL) and the resulting solution was cooled to 0 °C. Powdered 4 Å molecular sieves (0.2 g) were added and the suspension was stirred for 30 min at 0 °C before *N*-iodosuccinimide (65 mg, 0.29 mmol) and silver triflate (10 mg, 0.04 mmol) were added. The reaction mixture was stirred for 25 min, neutralized with Et_3_N, diluted with CH_2_Cl_2_ (10 mL) and filtered through Celite. The filtrate was washed successively with a saturated aqueous Na_2_S_2_O_3_ and water before being dried (Na_2_SO_4_), filtered and concentrated. The crude residue was purified by chromatography (3:1 hexanes–EtOAc) to afford 10 (0.41 g, 90%) as a white foam. [α]_D_ +7.0 (*c* = 0.3, CHCl_3_); *R_f_* 0.17 (3:1 hexanes–EtOAc); ^1^H NMR (500 MHz, CDCl_3_, δ_H_) 8.10–7.85 (m, 14H), 7.63–7.58 (m, 4 H), 7.55–7.20 (m, 27H), 5.81–5.76 (m, 2 H), 5.63 (dd, *J* = 5.7, 1.9 Hz, 1 H), 5.58 (d, *J* = 2.0 Hz, 1 H), 5.52 (d, *J* = 1.8 Hz, 1 H), 5.51–5.48 (m, 1 H), 5.44 (dd, *J* = 5.2, 1.5 Hz, 1 H), 5.39 (d, *J* = 1.5 Hz, 1 H), 5.24 (s, 1 H), 5.21 (s, 1 H), 4.80–4.76 (m, 1 H), 4.71 (dd, *J* = 11.9, 3.7 Hz, 1 H), 4.65–4.60 (m, 2 H), 4.51 (dd, *J* = 5.5, 3.7 Hz, 1 H), 4.49–4.45 (m, 1 H), 4.15–4.10 (m, 1 H), 3.94–3.86 (m, 3 H), 3.65 (ddd, *J* = 9.7, 6.6, 6.6 Hz, 1 H), 3.40 (ddd, *J* = 9.6, 6.5, 6.5 Hz, 1 H), 2.66–2.60 (m, 2 H), 2.60–2.50 (m, 2 H), 2.06 (s, 3H), 1.62–1.40 (m, 2 H), 1.30–1.18 (m, 10H), 0.96 (s, 9 H), 0.86 (dd, *J* = 6.9 Hz, 3 H); ^13^C NMR (125 MHz, CDCl_3_, δ_C_) 205.9, 172.0, 166.1, 165.7(3), 165.7, 165.4, 165.3, 165.1, 135.5(0), 135.5, 133.8, 133.3, 133.2((9), 133.2(5), 133.2(1), 133.2, 133.0, 129.9, 129.8(9), 129.8(5), 129.8(1), 129.8, 129.7, 129.3(4), 129.3(2), 129.2, 129.1(3), 129.1(1), 129.1, 128.4(2), 128.4, 128.3(4), 128.3(3), 128.3(1), 128.3, 128.2, 127.7(1), 127.7(0), 106.9, 105.9, 105.4, 82.7, 82.5, 82.0, 81.8, 80.8, 80.4,77.6, 77.3, 77.1, 77.0, 76.8, 75.5, 72.6, 67.7, 66.9, 63.6, 62.4, 38.0, 31.8, 29.7, 29.4(1), 29.4, 29.2, 28.6, 28.0, 26.6, 26.1, 22.6, 19.1, 14.0. HRMS (ESI) *m/z* calcd for (M + Na) C_95_H_98_O_24_SiNa: 1673.6109. Found: 1673.6109.

#### Octyl 2,3-di-*O*-benzoyl*-*6*-O-t-*butyldiphenylsilyl*-*β-D-galactofuranosyl-(1 → 6)-2,3-di-*O*-benzoyl-[5-*O*-(2,3,5-tri-*O*-benzoyl*-*α-D-arabinofuranosyl-)]-β-D-galactofuranoside (11)

A solution of 10 (0.39 g, 0.24 mmol) and hydrazine monohydrate–HOAc (0.6 mL 1:2) in CH_2_Cl_2_–CH_3_OH (9:1, 15 mL) was stirred for 75 min. The solvent was removed (< 20 °C) and the resulting oil was diluted with EtOAc (20 mL). The solution was washed with a saturated aqueous NaHCO_3_ (10 mL × 2) and brine (10 mL), dried (Na_2_SO_4_), filtered and concentrated. The crude residue was purified by chromatography (4:1 hexanes–EtOAc) to afford 11 (0.33 g, 91%) as a foam. [α]_D_ +4.0 (*c* = 0.36, CHCl_3_); *R_f_* 0.31 (3:1 hexanes–EtOAc); ^1^H NMR (500 MHz, CDCl_3_, δ_H_) 8.12–7.84 (m, 14H), 7.64–7.62 (m, 4 H), 7.58–7.20 (m, 27H), 5.84–5.76 (m, 2 H), 5.66–5.64 (m, 2 H), 5.61 (d, *J* = 1.9 Hz, 1 H), 5.56 (d, *J* = 1.8 Hz, 1 H), 5.43 (d, *J* = 1.5 Hz, 1 H), 5.25 (s, 1 H), 5.21 (s, 1 H), 4.81–4.72 (m, 2 H), 4.64 (dd, *J* = 11.9, 4.6 Hz, 1 H), 4.53 (dd, *J* = 5.6, 3.7 Hz, 1 H), 4.51–4.46 (m, 2 H), 4.16–4.10 (m, 2 H), 3.90–3.76 (m, 3 H), 3.66 (ddd, *J* = 9.6, 6.6, 6.6 Hz, 1 H), 3.40 (ddd, *J* = 9.6, 6.5, 6.5 Hz, 1 H), 2.50 (br. s, 1 H), 1.60–1.42 (m, 2 H), 1.35–1.20 (m, 10H), 1.00 (s, 9 H), 0.86 (dd, *J* = 6.9 Hz, 3 H); ^13^C NMR (125 MHz, CDCl_3_, δ_C_) 166.1, 165.7(8), 165.7(6), 165.6(8), 165.6(6), 165.3, 165.1, 135.5(2), 135.5(0), 133.3(3), 133.3(1), 133.2(9), 133.2(7), 133.2, 133.1(18), 133.1(10), 132.9, 129.9(4), 129.9(0), 129.8(9), 129.8(7), 129.8(6), 129.7(4), 129.7(3), 129.3(4), 129.3(1), 129.2, 129.1(2), 129.1(0), 128.5, 128.3(8), 128.3(5), 128.3, 128.2, 127.7, 106.9, 106.0, 105.5, 82.7, 82.6, 82.5, 82.0, 81.7, 80.4, 77.8, 77.7, 77.3, 77.2, 77.0, 76.8, 75.6, 71.0, 67.8, 66.8, 65.1, 63.6, 31.8, 29.4(1), 29.4, 29.2, 26.8, 26.1, 22.6, 19.2, 14.1. HRMS (ESI) *m/z* calcd for (M + Na) C_90_H_92_O_22_SiNa: 1575.5741(8). Found: 1575.5741(3).

#### Octyl 2,3,5,6-tetra-*O*-benzoyl*-*β-D-galactofuranosyl-(1 → 5)-2,3-di-*O*-benzoyl*-*6*-O-t-*butyl-diphenylsilyl*-*β-D-galactofuranosyl-(1 → 6)-2,3-di-*O*-benzoyl-[5-*O*-(2,3,5-tri-*O*-benzoyl*-*α-D-arabinofuranosyl-)]-β-D-galactofuranoside (13)

Thioglycoside 12 (Completo and Lowary, 2008) (0.18 g, 0.26 mmol) and alcohol 11 (0.32 g, 0.21 mmol) were dried over phosphorus pentoxide under vacuum for 6 h and then dissolved in CH_2_Cl_2_ (12 mL) and the resulting solution was cooled to 0 °C. Powdered 4 Å molecular sieves (0.2 g) were added and the suspension was stirred for 30 min at 0 °C before *N*-iodosuccinimide (75 mg, 0.33 mmol) and silver triflate (10 mg, 0.04 mmol) were added. The reaction mixture was stirred for 25 min, neutralized with Et_3_N, diluted with CH_2_Cl_2_ (15 mL) and filtered through Celite. The filtrate was washed successively with a saturated aqueous Na_2_S_2_O_3_ and water before being dried (Na_2_SO_4_), filtered and concentrated. The crude residue was purified by chromatography (3:1hexanes–EtOAc) to afford 13 (0.39 g, 89%) as a white foam. *R_f_* 0.17 (3:1hexanes–EtOAc); ^1^H NMR (500 MHz, CDCl_3_, δ_H_) 8.15–7.70 (m, 22H), 7.65–7.60 (m, 4H), 7.60–7.10 (m, 39H), 6.06 (ddd, *J* = 7.5, 3.6, 3.6 Hz, 1H), 5.86–5.78 (m, 3H), 5.76 (s, 1H), 5.69–5.66 (m, 2 H), 5.64 (dd, *J* = 5.2, 1.4 Hz, 1H), 5.60 (d, *J* = 2.0 Hz, 1H), 5.52 (d, *J* = 1.7 Hz, 1H), 5.44 (d, *J* = 1.8 Hz, 1H), 5.23 (s, 1H), 5.16 (s, 1H), 5.11 (dd, *J* = 5.2, 3.7 Hz, 1H), 4.88–4.82 (m, 2 H), 4.77–4.70 (m, 2 H), 4.66 (dd, *J* = 12.0, 4.5 Hz, 1H), 4.57–4.44 (m, 4H), 4.16–4.06 (m, 2 H), 3.98 (dd, *J* = 10.8, 5.4 Hz, 1H), 3.90–3.85 (m, 1H), 3.63 (ddd, *J* = 9.6, 6.5, 6.5 Hz, 1H), 3.37 (ddd, *J* = 9.6, 6.5, 6.5 Hz, 1H), 1.62–1.40 (m, 2 H), 1.40–1.18 (m, 10H), 0.96 (s, 9 H), 0.86 (dd, *J* = 6.9 Hz, 3H); ^13^C NMR (125 MHz, CDCl_3_, δ_C_) 176.4, 166.1, 166.0, 165.7(1), 165.7, 165.6, 165.5, 165.2(9), 165.2(7), 165.1, 140.9, 135.5, 133.8, 133.3, 133.2(0), 133.2, 133.1, 133.0(0), 133.0, 132.9, 130.4, 130.1, 130.0(2), 130.0, 129.9(3), 129.9(0), 129.9, 129.8, 129.7(2), 129.7, 129.6(4), 129.6, 129.4(0), 129.4, 129.3, 129.2, 129.1, 129.0, 128.8(4), 128.8, 128.5, 128.4, 128.3(3), 128.3(0), 128.2(8), 128.2(7), 128.2(1), 128.2, 128.1, 127.8, 127.7, 106.7, 106.0, 105.4, 105.1, 82.8, 82.4, 82.1(6), 82.1, 82.0, 81.8, 80.2, 77.9, 77.6, 77.3, 77.2, 77.1, 76.9, 76.8, 75.3, 74.5, 70.6, 67.6, 67.0, 64.0, 63.9, 63.6, 31.8, 29.4, 29.3, 28.6, 26.7, 26.1, 22.7, 21.4, 19.0, 14.1.

#### Octyl 2,3,5,6-tetra-*O*-benzoyl*-*β-D-galactofuranosyl-(1 → 5)-2,3-di-*O*-benzoyl*-*β-D-galactofuranosyl-(1 → 6)-2,3-di-*O*-benzoyl-[5-*O*-(2,3,5-tri-*O*-benzoyl*-*α-D-arabinofuranosyl-)]-β-D-galactofuranoside (14)

Compound 13 (0.39 g, 0.18 mmol) was dissolved in a solution of pyridine–THF (4:1, 12 mL) at 0 °C and 70% HF–pyridine (0.4 mL) was added dropwise. The reaction mixture was stirred for 48 h while warming to room temperature before being diluted with EtOAc (25 mL), poured into a saturated aqueous NaHCO_3_ (25 mL) and extracted with EtOAc. The organic layer was washed with water, dried (Na_2_SO_4_), filtered and concentrated to give crude syrup that was purified by column chromatography (2:1, hexanes–EtOAc) to afford 14 (0.32 g, 91%) as a white foam. [α]_D_ −7.9 (*c* = 0.22, CHCl_3_); *R_f_* 0.17 (7:3 hexanes–EtOAc); ^1^H NMR (500 MHz, CDCl_3_, δ_H_) 8.10–7.80 (m, 22H), 7.57–7.20 (m, 31H), 7.16–7.10 (m, 2 H), 6.04 (ddd, *J* = 7.4, 3.6, 3.6 Hz, 1 H), 5.86–5.83 (m, 2 H), 5.78 (dd, *J* = 5.0, 1.7 Hz, 1 H), 5.72 (s, 1 H), 5.69–5.66 (m, 2 H), 5.61 (s, 2 H), 5.51 (d, *J* = 1.7 Hz, 1 H), 5.45 (d, *J* = 1.7 Hz, 1 H), 5.31–5.22 (m, 2 H), 5.04 (dd, *J* = 5.4, 3.5 Hz, 1 H), 4.87–4.76 (m, 3 H), 4.72–4.66 (m, 2 H), 4.58–4.50 (m, 3 H), 4.36–4.32 (m, 1 H), 4.16–4.11 (m, 1 H), 4.01–3.96 (m, 2 H), 3.92–3.86 (m, 1 H), 3.74 (ddd, *J* = 9.6, 6.6, 6.6 Hz, 1 H), 3.48 (ddd, *J* = 9.6, 6.5, 6.5 Hz, 1 H), 2.81 (br.s, 1 H), 1.61–1.45 (m, 2 H), 1.40–1.20 (m, 10H), 0.86 (dd, *J* = 6.9 Hz, 3 H); ^13^C NMR (125 MHz, CDCl_3_, δ_C_) 166.1, 166.0, 165.9, 165.8, 165.7(0), 165.7, 165.6(2), 165.6, 165.3, 133.4(0), 133.4, 133.3(4), 133.3, 133.1, 133.0, 132.9, 130.0(8), 130.0(7), 130.0(4), 129.9(8), 129.9(6), 129.9(0), 129.9, 129.7(3), 129.6(9), 129.6(5), 129.6, 129.3, 129.2(4), 129.2(1), 129.2, 128.9, 128.8(4), 128.8(1), 128.7, 128.5, 128.4(0), 128.3(8), 128.3(6), 128.3(3), 128.3, 128.2(3), 128.2, 106.7, 106.0, 105.4, 105.2, 82.9, 82.8, 82.7, 82.5, 82.4, 82.0, 81.7, 80.5, 77.8, 77.6, 77.4, 77.3, 77.0(9), 77.0(5), 76.9, 76.8, 75.7, 74.5, 70.5, 67.8, 67.0, 63.8, 63.6, 61.9, 31.8, 29.4(4), 29.4(1), 29.3, 26.1, 22.6, 14.1. HRMS (ESI) *m/z* calcd for (M + Na) C_108_H_100_O_31_Na: 1915.6140. Found: 1915.6150.

#### Octyl β-D-galactofuranosyl-(1→5)-β-D-galactofuranosyl-(1→6)-β-D-[5-*O*-(α-D-arabinofuranosyl-)]-galactofuranoside (MJ-13-77)

To a solution of 14 (0.31 g, 0.16 mmol) in CH_2_Cl_2_–CH_3_OH (2:1, 12 mL) was added 1 M NaOCH_3_ (0.25 mL). The reaction mixture was stirred for 48 h with occasional addition of CH_3_OH (3 mL × 4) and was neutralized with the careful addition of Amberlyst-IR-120 (H+) cation exchange resin. The solution was filtered and concentrated to give a syrup that was dissolved in distilled water (10 mL). The aqueous phase was washed with EtOAc (3 mL × 2) and CH_2_Cl_2_ (5 mL) and then lyophilized to give MJ-13-77 (0.12 g, quantitative) as a fluffy solid: [α]_D_ −66.5 (*c* = 0.2, CH_3_OH); *R_f_* 0.39 (7:3:0.1 CH_2_Cl_2_–CH_3_OH–H_2_O); ^1^H NMR (500 MHz, D_2_O, δ_H_) 5.22–5.18 (m, 2H), 4.98 (d, *J* = 1.5 Hz, 1H), 4.95 (d, *J* = 1.8 Hz, 1H), 4.16–3.85 (m, 15H), 3.85–3.61 (m, 9H), 3.55 (ddd, *J* = 10.0, 6.6, 6.6 Hz, 1H), 1.62–1.56 (m, 2H), 1.40–1.20 (m, 10H), 0.85 (dd, *J* = 6.5 Hz, 3H); ^13^C NMR (125 MHz, CDCl_3_, δ_C_) 109.5 (C-1), 108.5 (C-1), 108.0 (C-1), 107.7 (C-1), 84.7, 83.5, 82.7, 82.1, 82.0, 77.6, 77.4, 77.3, 77.1, 76.7, 71.4, 69.5, 68.3, 63.7, 62.1, 62.0, 32.1, 29.6, 29.4, 26.2, 23.0, 14.4. HRMS (ESI) *m/z* calcd for (M + Na) C_31_H_56_O_20_Na: 771.3257. Found: 771.3248.

#### *p*-Thiotolyl 2,3-di-*O*-benzoyl*-*β-D-galactofuranoside (16)

To a solution of compound 15 (Completo and Lowary, 2008) (0.99 g, 3.5 mmol) in acetone (40 mL) and 2,2-dimethoxypropane (20 mL) at room temperature was added *p*-TSA·H_2_O (13 mg, 0.07 mmol) and the solution was stirred 90 min. The reaction was then quenched by adding four drops of triethylamine and the mixture was concentrated on a rotary evaporator. The syrupy residue was then dissolved in pyridine (10 mL) and cooled to 0 °C followed by the addition of benzoyl chloride (1.3 mL, 11.2 mmol) dropwise. The reaction mixture was allowed to warm to room temperature and stirred overnight. The reaction was quenched by the addition of chilled water (50 mL) and extracted with CH_2_Cl_2_ (60 mL). The CH_2_Cl_2_ layer was washed with 10% aqueous copper sulfate solution (30 mL × 4), water (40 mL). The separated CH_2_Cl_2_ layer was dried (Na_2_SO_4_) and concentrated. The syrupy residue obtained was dissolved in a solution of acetic acid: water: THF (3:1.5:1.5; 60 mL) and heated at 55–60 °C for 9 h. The reaction mixture was then directly concentrated on rotary evaporator at 50 °C and the residue was purified by column chromatography (3:2hexanes–EtOAc) to yield 16 (0.96 g, 56% over three steps) as a thick syrup. [α]_D_ −77.8 (*c* = 0.9, CHCl_3_); *R_f_* 0.26 (3:2hexanes–EtOAc); ^1^H NMR (500 MHz, CDCl_3,_ δ_H_) 8.15–8.10 (m, 2H), 8.10–8.05 (m, 2H), 7.65–7.55 (m, 2H), 7.55–7.40 (m, 6H), 7.16–7.12 (m, 2H), 5.72–5.66 (m, 3H, H-1, H-2, H-3), 4.58 (dd, *J* = 4.8, 3.4 Hz, 1H, H-4), 4.19–4.14 (m, 1H, H-5), 3.85 (dd, *J* = 11.6, 5.6 Hz, 1H, H-6), 3.80 (dd, *J* = 11.6, 4.6 Hz, 1H, H-6′), 2.81 (br.s, 2H), 2.35 (s, 3H); ^13^C NMR (125 MHz, CDCl_3_, δ_C_) 166.0, 138.4, 133.7(2), 133.7, 133.2, 130.1, 129.9, 129.4, 128.9, 128.6, 91.8, 84.1, 81.8, 78.1, 70.5, 64.3, 21.2. HRMS (ESI) *m/z* calcd for (M + Na) C_27_H_26_O_7_SNa: 517.1291. Found: 517.1294.

#### *p*-Thiotolyl 2,3-di-O-benzoyl-6-O-t-butyldiphenylsilyl-β-D-galactofuranoside (17)

To a solution of 16 (0.92 g, 1.9 mmol) in pyridine (15 mL) and CH_2_Cl_2_ (10 mL) at 0 °C was added *t*-butyldiphenylsilyl chloride (0.76 mL, 3.0 mmol) dropwise. The solution was then stirred overnight with warming to room temperature before CH_3_OH (0.4 mL) was added. After stirring for 30 min, the solution was poured into a saturated aqueous NaHCO_3_ (20 mL) and then extracted with CH_2_Cl_2_ (30 mL). The organic layer was washed with brine, dried (Na_2_SO_4_), filtered and concentrated to a residue that was purified by chromatography (85:15 hexanes–EtOAc) to yield 17 (1.3 g, 95%) as a thick syrup. [α]_D_ −53.0 (*c* = 0.8, CHCl_3_); *R_f_* 0.38 (4:1hexanes–EtOAc); ^1^H NMR (500 MHz, CDCl_3_, δ_H_) 8.17–8.02 (m, 4H), 7.70–7.52 (m, 6H), 7.52–7.40 (m, 8H), 7.40–7.30 (m, 4H), 7.09–7.05 (m, 2H), 5.77–5.67 (m, 3H, H-1, H-2, H-3), 4.77 (dd, *J* = 5.2, 2.4 Hz, 1H, H-4), 4.25–4.18 (m, 1H, H-5), 3.85 (dd, *J* = 10.2, 6.3 Hz, 1H, H-6), 3.77 (dd, *J* = 10.2, 6.7 Hz, 1H, H-6), 2.45 (d, *J* = 7.6 Hz, 1H), 2.33 (s, 3H), 1.06 (s, 9H); ^13^C NMR (125 MHz, CDCl_3_, δ_C_) 165.7, 165.3, 138.0, 135.6, 133.5, 133.2, 133.1(1), 133.1, 130.0, 129.9, 129.8, 129.2, 129.1, 128.5, 127.8, 91.7, 82.0, 78.1, 70.8, 64.7, 26.8, 21.2, 19.2. HRMS (ESI) *m/z* calcd for (M + Na) C_43_H_44_O_7_SiSNa: 755.2469. Found: 755.2470.

#### Octyl 2,3-di-*O*-benzoyl-5-*O*-*t*-butyldiphenylsilyl-β-D-galactofuranosyl-(1→5)-2,3,6-tri-*O*-benzoyl-β-D-galactofuranoside (20)

Alcohol 18 (Completo and Lowary, 2008) (0.37 g, 0.6 mmol) was glycosylated with thioglycoside 9 (0.6 g, 0.7 mmol) using in *N*-iodosuccinimide (0.17 g, 0.75 mmol) and silver triflate (0.02 g, 0.08 mmol) in CH_2_Cl_2_ (20 mL) containing powdered 4 Å molecular sieves (0.25 g) as described for the preparation of 10. The crude product 19 obtained was used directly for the next step as follows. A solution of crude 19 from above and hydrazine monohydrate–HOAc (0.5 mL 1:2) in CH_2_Cl_2_–CH_3_OH (9:1, 20 mL) was stirred for 90 min. The solvent was removed (<20 °C) and the resulting oil was diluted with EtOAc (30 mL). The solution was washed with a saturated aqueous NaHCO_3_ (15 mL × 2) and brine (15 mL), dried (Na_2_SO_4_), filtered and concentrated. The crude residue was purified by chromatography (4:1hexanes–EtOAc) to afford 20 (0.63 g, 84% over two steps) as a foam. [α]_D_ −1.7 (*c* = 0.21, CHCl_3_); *R_f_* 0.32 (4:1hexanes–EtOAc); ^1^H NMR (500 MHz, CDCl_3_, δ_H_) 8.05–7.82 (m, 10H), 7.62–7.20 (m, 25H), 5.80–5.76 (m, 1H), 5.72 (s, 1H), 5.71–5.68 (m, 2H), 5.47 (d, *J* = 1.4 Hz, 1H), 5.13 (s, 1H), 4.77–4.72 (m, 1H), 4.70–4.60 (m, 3H), 4.49 (dd, *J* = 5.0, 3.8 Hz, 1H), 4.13–4.08 (m, 1H), 3.83–3.80 (m, 2H), 3.63 (ddd, *J* = 9.5, 6.7, 6.7 Hz, 1H), 3.41 (ddd, *J* = 9.5, 6.3, 6.3 Hz, 1H), 2.63 (br. s, 1H), 1.62–1.46 (m, 2H), 1.40–1.20 (m, 10H), 1.00 (s, 9H), 0.87 (dd, *J* = 6.9 Hz, 3H); ^13^C NMR (125 MHz, CDCl_3_, δ_C_) 166.1, 165.7, 165.6, 165.4, 165.2, 135.5, 133.4, 133.3, 133.2, 133.1, 133.0, 129.9, 129.8, 129.6, 129.2, 129.1, 128.5, 128.4, 128.3, 128.2, 127.7, 105.5, 82.9, 82.3, 81.9, 81.7, 77.9, 77.3, 77.1, 77.0, 76.8, 73.3, 71.5, 67.6, 65.3, 64.6, 31.8, 29.5, 29.4, 26.8, 26.1, 22.7, 19.2. HRMS (ESI) *m/z* calcd for (M + Na) C_71_H_76_O_16_SiNa: 1235.4794. Found: 1235.4794.

#### Octyl 2,3,5-tri-*O*-benzoyl-α-D-arabinofuranosyl-(1 → 5)-2,3-di-*O*-benzoyl-5-*O*-*t*-butyl-diphenylsilyl-β-D-galactofuranosyl-(1 → 5)-2,3,6-tri-*O*-benzoyl-β-D-galactofuranoside (21)

Alcohol 20 (0.21 g, 0.17 mmol) was glycosylated with thioglycoside 6 (Joe et al., 2011) (0.12 g, 0.21 mmol) using in *N*-iodosuccinimide (0.065 g, 0.29 mmol) and silver triflate (0.01 g, 0.04 mmol) in CH_2_Cl_2_ (8 mL) containing powdered 4 Å molecular sieves (0.15 g) as described for the preparation of 10. The crude residue was purified by chromatography (4:1 hexanes–EtOAc) to afford 21 (0.27 g, 93%) as a white foam. *R_f_* 0.24 (4:1 hexanes–EtOAc); ^1^H NMR (500 MHz, CDCl_3_, δ_H_) 8.20–7.89 (m, 12H), 7.85–7.75 (m, 4H), 7.61–7.05 (m, 34H), 5.89 (d, *J* = 4.9 Hz, 1 H), 5.81 (s, 1 H), 5.74–5.66 (m, 3 H), 5.57 (d, *J* = 4.7 Hz, 1 H), 5.49 (s, 1 H), 5.43 (d, *J* = 1.3 Hz, 1 H), 5.15 (s, 1 H), 4.93 (dd, *J* = 5.4, 5.4 Hz, 1 H), 4.81–4.56 (m, 5 H), 4.53–4.46 (m, 2 H), 4.42 (q, *J* = 5.7 Hz, 1 H), 3.98 (dd, *J* = 10.7, 5.6 Hz, 1 H), 3.94 (dd, *J* = 10.7, 6.1 Hz, 1 H), 3.68 (ddd, *J* = 9.6, 6.8 Hz, 1 H), 3.44 (ddd, *J* = 9.6, 6.3 Hz, 1 H), 1.62–1.52 (m, 2 H), 1.40–1.20 (m, 10H), 0.96 (s, 9 H), 0.86 (dd, *J* = 6.9 Hz, 3 H); ^13^C NMR (125 MHz, CDCl_3_, δ_C_) 166.2, 166.1, 165.7, 165.6(0), 165.6, 165.5, 165.2, 165.1, 135.5, 135.4, 133.3(0), 133.3, 133.2(2), 133.2, 133.1(1), 133.1, 133.0, 132.9(4), 132.9, 129.9(0), 129.9, 129.8(0), 129.8, 129.7(4), 129.7, 129.6, 129.2, 129.1(3), 129.1(1), 129.1, 128.5, 128.4, 128.3(3), 128.3(1), 128.2(8), 128.2(6), 128.1, 127.7, 127.6, 105.7, 105.5, 104.7, 82.6(3), 82.6, 82.5, 82.2, 81.7, 80.7, 77.7(1), 77.7, 77.3, 77.2, 77.1, 76.8, 76.7, 72.4, 67.5, 64.6, 63.6, 63.4, 31.8, 29.5, 29.4, 29.3, 26.8, 26.1, 22.7, 19.1, 14.1.

#### Octyl 2,3,5-tri-*O*-benzoyl-α-D-arabinofuranosyl-(1→5)-2,3-di-*O*-benzoyl-β-D-galactofuranosyl-(1→5)-2,3,6-tri-*O*-benzoyl-β-D-galactofuranoside (22)

To a solution of compound 21 (0.27 g, 0.19 mmol) in THF–pyridine (4:1, 9 mL) at 0 °C was added 70% HF·pyridine (0.4 mL) dropwise. The solution was warmed to room temperature and stirred for 36 h before being poured into a saturated aqueous NaHCO_3_ (20 mL), extracted with CH_2_Cl_2_ (25 mL) and washed with brine (20 mL). The organic layer was then dried (Na_2_SO_4_), filtered and concentrated to a syrup that was purified by chromatography (7:3 hexanes–EtOAc) to yield 22 (0.21 g, 88%) as a thick syrup. [α]_D_ −7.0 (*c* = 0.2, CHCl_3_); *R_f_* 0.26 (7:3 hexanes–EtOAc); ^1^H NMR (500 MHz, CDCl_3_, δ_H_) 8.10–7.95 (m, 12H), 7.95–7.85 (m, 2 H), 7.85–7.80 (m, 2 H), 7.58–7.20 (m, 24H), 5.84 (dd, *J* = 5.2, 1.3 Hz, 1 H), 5.80 (dd, *J* = 6.1, 2.3 Hz, 1 H), 5.78–5.73 (m, 3 H), 5.63 (dd, *J* = 5.1, 2.1 Hz, 1 H), 5.58 (d, *J* = 2.1 Hz, 1 H), 5.49 (d, *J* = 1.4 Hz, 1 H), 5.25 (s, 1 H), 4.78–4.68 (m, 5 H), 5.68–4.61 (m, 2 H), 4.54 (dd, *J* = 5.1, 3.7 Hz, 1 H), 4.28–4.22 (m, 1 H), 3.96–3.86 (m, 2 H), 3.72 (ddd, *J* = 9.5, 6.7, 6.7 Hz, 1 H), 3.50 (ddd, *J* = 9.5, 6.3, 6.3 Hz, 1 H), 3.17 (dd, *J* = 7.7, 5.3 Hz, 1 H), 1.62–1.52 (m, 2 H), 1.41–1.20 (m, 10H), 0.84 (dd, *J* = 6.9 Hz, 3 H); ^13^C NMR (125 MHz, CDCl_3_, δ_C_) 166.1(4), 166.1(2), 166.1, 165.7, 165.6, 165.5, 165.2, 133.5(1), 133.5, 133.4, 133.3, 133.2(3), 133.2, 133.1, 133.0, 129.9(1), 129.8(9), 129.8(7), 129.8(5), 129.8(1), 129.8, 129.6(4), 129.6(2), 129.6, 129.1, 129.0(3), 128.9(9), 128.9(6), 128.5, 128.4(2), 128.4(0), 128.4, 128.3, 128.2, 107.3, 105.5(4), 105.5, 82.5, 82.3, 82.2, 81.8, 80.7, 78.8, 77.4(4), 77.4(2), 77.3(4), 77.3, 77.0, 76.8, 73.6, 67.6, 64.7, 63.8(4), 62.8(2), 31.8, 29.5, 29.4, 29.3, 26.2, 22.7, 14.1. HRMS (ESI) *m/z* calcd for (M + Na) C_81_H_78_O_23_Na: 1441.4826. Found: 1441.4823.

#### Octyl 2,3,5,6-tetra-*O*-benzoyl*-*β-D-galactofuranosyl-(1 → 6)-2,3-di-*O*-benzoyl-[5-*O*-(2,3,5-tri-*O*-benzoyl-α-D-arabinofuranosyl-)]-β-D-galactofuranosyl-(1 → 5)-2,3,6-tri-*O*-benzoyl-β-D-galactofuranoside (23)

Alcohol 22 (0.2 g, 0.14 mmol) was glycosylated with thioglycoside 12 (Completo and Lowary, 2008) (0.12 g, 0.17 mmol) using in *N*-iodosuccinimide (0.05 g, 0.22 mmol) and silver triflate (0.006 g, 0.02 mmol) in CH_2_Cl_2_ (7 mL) containing powdered 4 Å molecular sieves (0.1 g) as described for the preparation of 10. The crude residue was purified by chromatography (2:1hexanes–EtOAc) to afford 23 (0.25 g, 90%) as a white foam. [α]_D_ −0.5 (*c* = 0.2, CHCl_3_); *R_f_* 0.27 (7:3 hexanes–EtOAc); ^1^H NMR (500 MHz, CDCl_3_, δ_H_) 8.06–7.92 (m, 18H), 7.84–7.78 (m, 4H), 7.70–7.66 (m, 2H), 7.50–7.12 (m, 36H), 5.99 (ddd, *J* = 5.7, 3.3 Hz, 1H), 5.91 (dd, *J* = 5.1, 1.3 Hz, 1H), 5.84 (s, 1H), 5.82–5.76 (m, 2H), 5.72 (d, *J* = 1.9 Hz, 1H), 5.65–5.60 (m, 2H), 5.50 (d, *J* = 1.3 Hz, 1H), 5.45 (d, *J* = 1.5 Hz, 1H), 5.42 (d, *J* = 1.5 Hz, 1H), 5.27 (s, 1H), 5.21 (s, 1H), 4.90–4.84 (m, 2H), 4.82–4.60 (m, 8 H), 4.59–4.54 (m, 2H), 4.15 (dd, *J* = 10.8, 4.1 Hz, 1H), 3.86 (dd, *J* = 10.8, 7.0 Hz, 1H), 3.71 (ddd, *J* = 9.6, 6.7, 6.7 Hz, 1H), 3.47 (ddd, *J* = 9.6, 6.3, 6.3 Hz, 1H), 1.65–1.50 (m, 2H), 1.40–1.20 (m, 10H), 0.83 (dd, *J* = 6.9 Hz, 3 H); ^13^C NMR (125 MHz, CDCl_3_, δ_C_) 166.1, 166.0, 165.6, 165.5, 165.3, 165.1(2), 165.1, 133.3, 133.2, 133.1, 132.9, 132.8, 129.9, 129.8, 129.7, 129.6(3), 129.6, 129.2, 129.1, 129.0, 128.9, 128.8, 128.5, 128.4, 128.3, 128.1, 106.5, 106.0, 105.5, 104.9, 82.8, 82.7, 82.5, 82.2, 81.8, 81.4, 80.7, 77.6, 77.4, 77.2, 75.2, 72.8, 70.3, 67.9, 67.5, 64.6, 63.7, 63.5, 31.8, 29.6, 29.4, 29.3, 26.2, 22.6, 14.1. HRMS (ESI) *m/z* calcd for (M + Na) C_115_H_104_O_32_Na: 2019.6402. Found: 2019.6382.

#### Octyl β-D-galactofuranosyl-(1→6)-β-D-[5-*O*-(α-D-arabinofuranosyl-)]-galactofuranosyl-(1→5)-β-D-galactofuranoside (MJ-14-01)

To a solution of 23 (0.24 g, 0.12 mmol) in CH_2_Cl_2_–CH_3_OH (2:1, 9 mL) was added 1 M NaOCH_3_ (0.25 mL). The reaction mixture was stirred for 24 h with occasional addition of CH_3_OH (3 mL × 4) and was neutralized with the careful addition of Amberlyst-IR-120 (H+) cation exchange resin. The solution was filtered and concentrated to give a syrup that was dissolved in distilled water (10 mL). The aqueous phase was washed with EtOAc (3 mL × 2) and CH_2_Cl_2_ (6 mL) and then lyophilized to give MJ-14-01 (0.09 g, quantitative) as a fluffy solid: [α]_D_ −62.5 (*c* = 0.23, CH_3_OH); *R_f_* 0.33 (7:3:0.1 CH_2_Cl_2_–CH_3_OH–H_2_O); ^1^H NMR (500 MHz, D_2_O, δ_H_) 5.21 (d, *J* = 1.7 Hz, 2H), 5.02 (d, *J* = 1.9 Hz, 1H), 4.95 (d, *J* = 2.3 Hz, 1H), 4.20 (dd, *J* = 6.6, 5.4 Hz, 1H), 4.17–4.07 (m, 5H), 4.07–3.96 (m, 6H), 3.96–3.90 (m, 3H), 3.85–3.67 (m, 8H), 3.67–3.60 (m, 1H), 3.56 (ddd, *J* = 10.0, 6.5, 6.5 Hz, 1H), 1.64–1.56 (m, 2H), 1.39–1.20 (m, 10H), 0.85 (dd, *J* = 6.5 Hz, 3H); ^13^C NMR (125 MHz, CDCl_3_, δ_C_) 109.4 (C-1), 108.6 (C-1), 107.9 (C-1), 107.7 (C-1), 84.7, 83.7, 83.4, 82.3, 82.2, 82.0(3), 82.0, 81.9, 77.6, 77.5, 77.4, 77.3, 76.9, 71.6, 69.5, 68.6, 63.7, 62.0, 32.0, 29.5, 29.3, 26.1, 22.9, 14.3. HRMS (ESI) *m/z* calcd for (M + Na) C_31_H_56_O_20_Na: 771.3257. Found: 771.3247.

### Bacterial strains and growth conditions

*C. glutamicum* ATCC 13032 (the wild type strain referred for the remainder of the text as *C. glutamicum*) and the recombinant strains were cultivated at 30 °C in either a rich BHI medium (Difco) or a salt medium CGXII as described previously (Eggeling and Bott, 2005). Samples for cell wall analysis were prepared by harvesting cells at an optical density of 10–15 followed by a saline wash and freeze drying.

### Construction of plasmids and strains

In order to generate the double deletion mutant *C. glutamicumΔaftAΔemb*, the deletion vector pK19mobsacB*ΔaftA* (*NCgl0185*) was constructed as previously described (Alderwick et al., 2006a, Belanger et al., 1996) and introduced into the previously reported *C. glutamicumΔemb* (*ΔNCgl0184*) strain (Alderwick et al., 2005), to generate *C. glutamicumΔaftAΔemb*. The chromosomal deletion of *NCgl0185* was achieved using two rounds of positive selection as described previously (Schäfer et al., 1994). A similar strategy was employed to successfully generate the double deletion mutant *C. glutamicumΔaftBΔaftD* (Alderwick et al., 2018a).

### Cell wall associated and cell wall bound lipid extraction and analysis

Cells were harvested and equivalent amounts of biomass (100 mg) were extracted using 2 ml of chloroform:methanol:water (10:10:3, v/v/v) for 4 h at 50 °C. The organic extracts were combined with 1.75 ml of chloroform and 0.75 ml of water. The lower organic phase containing associated lipids was recovered, washed with chloroform:methanol:water (3:47:48, v/v/v) and dried. Samples were resuspended in chloroform:methanol:water (10:10:3, v/v/v) and equivalent aliquots were subjected to thin-layer chromatography (TLC) analysis using silica gel plates (5554 silica gel 60F254, Merck). Alternatively, *C. glutamicum* cultures were metabolically labelled at mid-logarithmic phase of growth using 1 μCi ml^−1^ [1,2-^14^C]acetate (50–62 mCi mmol^−1^ (GE Healthcare) for 4 h at 30 °C with shaking, harvested and processed as described above. TLC plates were developed in chloroform:methanol:ammonium hydroxide (80:20:2, v/v/v) and cell wall associated lipids visualised with either molybdophosphoric acid (5% in ethanol; w/v) followed by heating or by autoradiography by exposure of Kodak BioMax MR film. In the latter case, labelled lipids were quantified by phosphorimaging and compared with known standards. The bound corynomycolic acids from the above delipidated extracts were released by addition of 2 ml of 5% (v/v) tetra-butyl ammonium hydroxide, followed by a 12 h incubation at 100 °C. After cooling, water (2 ml), dichloromethane (4 ml) and methyl iodide (100 μl) were added and mixed thoroughly for 30 min. The organic phase was recovered, washed repeatedly with water and resuspended in diethyl-ether. After centrifugation, the clear supernatant containing cell wall bound corynomycolic acid methyl esters (CMAMES) were dried and resuspended in dichloromethane. Equivalent aliquots were subjected to the TLC plates (5554 silica gel 60F254, Merck), developed in petroleum ether/acetone (95:5, v/v) and lipids visualised using molybdophosphoric acid (5% in ethanol; w/v) followed by heating or by autoradiography using Kodak BioMax MR film.

### Isolation of the mAGP complex and glycosyl composition of alditol acetates by gas chromatography

Cells were resuspended in phosphate-buffered saline containing 2% Triton X-100 (pH 7.2), disrupted by sonicaton and centrifuged. The pelleted material was extracted three times with 2% SDS in phosphate-buffered saline at 95 °C for 1 h, washed with water, 80% (v/v) acetone in water, and acetone, and subsequently lyophilised to yield a highly purified cell wall preparation (Besra et al., 1995). The mAGP preparations (5–10 mg) were hydrolysed using 2 M trifluoroacetic acid at 120 °C for 2 h and reduced using 100 μl of sodium borohydride solution (10 mg/ml resuspended in ethanol: 1 M ammonium hydroxide, 1:1). The obtained alditols were per-*O*-acetylated using 100 μl of acetic anhydride at 100 °C for 1 h before examination by gas chromatography (GC) as described previously (Alderwick et al., 2005, Besra et al., 1995).

### Isolation of AG and ^1^H/^13^C-nuclear magnetic resonance spectroscopy

The mAGP preparation was subjected to base saponification to remove mycolic acids using 2% potassium hydroxide in methanol-toluene (1:1) for 48 h. The insoluble residue was recovered by centrifugation at 27,000 g. The sample was washed repeatedly with methanol and the resulting AGP treated with 75 ml of 2 M sodium hydroxide for 16 h at 80 °C. The supernatant, which contained base-solubilised AG, was recovered by centrifugation at 27,000 g for 30 min. The crude AG preparation was neutralised with acetic acid and dialysed to remove residual salts (MWCO 3500). The supernatant was diluted in cold ethanol (80%, v/v) and left at −20 °C overnight to precipitate the base-solubilised AG, which was then recovered by centrifugation and lyophilised. Nuclear magnetic resonance (NMR) spectra of samples were recorded using Bruker DMX-500. Samples were repeatedly exchanged in deuterium oxide (99.9 atom% D) with intermediate lyophilisation and analysed at 313 K. The ^1^H and ^13^C NMR chemical shifts were referenced relative to internal acetone at 2.225 and 34.00 ppm, respectively. Details concerning NMR sequences used and experimental procedures were described previously (Daffé et al., 1990).

### Arabinofuranosyltransferase assays using p[^14^C]Rpp

Membrane and a P60 cell-free wall preparation were prepared to a final concentration of 10–15 mg/ml as described previously (Birch et al., 2008, Lee et al., 1997, Seidel et al., 2007). The branched tetrasaccharide neoglycolipid acceptors, β-D-Gal*f*-(1 → 5)-β-D-Gal*f*-(1 → 6)[α-D-Ara*f*-(1 → 5)]-β-D-Gal*f*-*O*-(CH2)7CH3 [**MJ-13-77**] and β-D-Gal*f*-(1 → 6)-[α-D-Ara*f*-(1 → 5)]-β-D-Gal*f*-(1 → 5)-β-D-Gal*f*-*O*-(CH2)7CH3 [**MJ-14**-**01**], 2 μl from a 20 mM stock solution and decaprenyl phosphate (1 µl of mg/ml) were aliquoted into 1.5 ml Eppendorf tubes and dried. IgePal™ (Sigma-Aldrich) was added (0.1%, v/v) with the appropriate amount of buffer (50 mM MOPS pH 7.9, 10 mM MgSO4, 5 mM β-mercaptoethanol) to a final volume of 80 μl. Samples were sonicated for 15 min to resuspend lipid-linked substrates and then mixed with the remaining assay components: membrane protein and ‘P60’ fraction (1 mg each) from either *C. glutamicum*, *C. glutamicumΔaftA*, *C. glutamicumΔemb*, *C. glutamicumΔaftAΔemb*, *C. glutamicumΔaftBΔaftD*, 1 mM ATP, 1 mM NADP, p[^14^C]Rpp (25,000 cpm) and in some cases EMB (1 mg/ml). Reaction mixtures were incubated for 1 h at 37 °C, quenched by the addition of 533 μl of chloroform/methanol (1:1, v/v) and mixed for 30 min. Supernatant was recovered following centrifugation at 27,000 g for 30 min and dried under nitrogen. The residue was resuspended in 750 μl ethanol:water (1:1, v/v) and loaded onto 1 ml SepPak ion exchange columns, pre-equilibrated with ethanol:water (1:1, v/v) as described previously (Lee et al., 1997). The column was washed twice with 2 ml of ethanol (100%) and the eluate collected and dried. The sample was resuspended in a mixture of water-saturated n-butanol (3 ml) and water (3 ml), mixed and the organic phase recovered following centrifugation. The aqueous phase was extracted once again with n-butanol (3 ml) and the organic phases pooled. The extracts were further washed using n-butanol-saturated water (3 ml). Finally, the n-butanol fraction was dried and resuspended in 200 μl of n-butanol. The incorporation of [^14^C]Ara*f* was determined by subjecting samples to TLC using silica gel plates (5735 silica gel 60F254, Merck) developed in isopropanol:acetic acid:water (8:1:1, v/v/v) and visualised by autoradiography employing Kodak BioMax MR films.

### Characterisation of *in vitro* reaction products generated by arabinofuranosyltransferases and *C. glutamicum* membranes

Large-scale reaction mixtures containing cold pRpp (25 mM, Sigma Aldrich) and neoglycolipid acceptors (**MJ-13**-**77** and **MJ-14**-**01**, 8 μl of 20 mM) were mixed and given an initial incubation of 1 h at 37 °C with membranes prepared from *C. glutamicum*. The assays were replenished with fresh membranes and re-incubated for 1 h at 37 °C with the entire process repeated thrice. Products were extracted from reaction mixtures by *n*-butanol:water phase separation and subjected to preparative TLC plates developed in isopropanol:acetic acid:water (8:1:1, v/v/v) as described above. Bands of interest were recovered from the plates by extraction with *n*-butanol. Samples were analysed by matrix-assisted laser desorption/ionisation-time-of-flight mass spectrometry (MALDI-TOF MS) and MS/MS as previously described (Lee et al., 1997, Shi et al., 2008).

## Results

### Comparison of Emb proteins and construction of a *C. glutamicumΔaftAΔemb* mutant

In previous studies EMB was shown to specifically inhibit AG biosynthesis, with the precise molecular target being the products of the *embCAB* loci in *M. tuberculosis* (Telenti et al., 1997) and *embRAB* loci in *M. avium* (Belanger et al., 1996). In comparison, *C. diphtheriae* and *C. glutamicum* have only one *emb* gene (Alderwick et al., 2005, Cerdeño-Tárraga et al., 2003, Kalinowski et al., 2003). This discrepancy is in accordance with the notion that corynebacteria have rarely undergone extensive genome rearrangements and have maintained ancestral genome structures even after the divergence of corynebacteria and mycobacteria, resulting in a low frequency of structural alterations and gene duplications (Alderwick et al., 2005, Nakamura et al., 2003). Interestingly, the single *emb* gene of *C. glutamicum* exhibits a higher identity to *embC* than to *embA* and *embB* of mycobacteria (Alderwick et al., 2005). Overexpression studies of the single *emb* gene from *C. glutamicum* resulted in an increased resistance of corynebacteria to EMB strongly suggesting that this front-line anti-TB drug inhibits Emb in *C. glutamicum* (Radmacher et al., 2005).

**Fig. 1:**
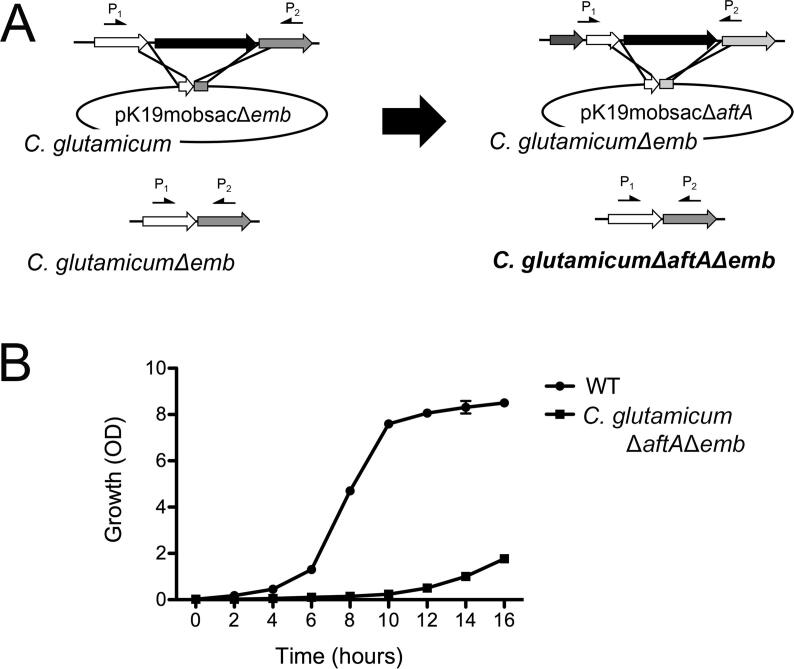
Construction and characterisation of the *C. glutamicumΔaftAΔemb* double mutant. (A) The *C. glutamicum emb* with its adjacent genes and the strategy to delete it using the deletion vector pK19mob*sacB*Δ*emb* as previously described (Alderwick et al., 2005). The deletion vector carries 12 nucleotides of the 5′-end of *emb* and 12 nucleotides of its 3′-end thus enabling the in-frame deletion of almost the entire *emb* gene. The arrows marked as P1 and P2 locate primers used for the PCR analysis to confirm the absence of *emb*. The *C. glutamicumΔemb* was then employed to subsequently generate *C. glutamicumΔaftAΔemb*. Previously reported deletion vector pK19mob*sacBΔaftA* was used to delete *aftA* in *C. glutamicumΔemb* (Alderwick et al., 2006b). Distances are not drawn to scale. (B) The consequences of both *aftA* and *emb* double deletion on growth of wild type *C. glutamicum* (WT).

We previously generated *C. glutamicumΔemb* (Fig. 1A) and *C. glutamicumΔaftA* mutants (Alderwick et al., 2005, Alderwick et al., 2006b), as well as the non-replicative vectors pK19mobsacBΔ*emb* and pK19mobsacBΔ*aftA* knock-out plasmids, carrying sequences adjacent to *emb* and *aftA,* respectively (Alderwick et al., 2006b, Alderwick et al., 2005). To generate the required double deletion mutant, *C. glutamicum aftA* was deleted in *C. glutamicumΔemb* using the pK19mobsacBΔ*aftA* plasmid, resulting in a double *ΔaftaΔemb* deletion mutant (Fig. 1A).

The growth characteristics of both *C. glutamicum* and *C. glutamicumΔaftAΔemb* were studied in brain heart infusion media supplemented with sorbitol for osmotic stabilisation (Eggeling and Bott, 2005). Optical density (OD) measurements revealed normal growth kinetics for the wild-type *C. glutamicum* and a severe reduction in growth rate for *C. glutamicumΔaftAΔemb*. Growth of *C. glutamicum* was completed after 10 h (OD ∼8.0), whereas *C. glutamicumΔaftAΔemb* scarcely reached an OD of 0.24 after 16 h (Fig. 1B).

### Characterisation of cell wall lipids from *C. glutamicum* and *C. glutamicumΔaftAΔemb*

**Fig. 2:**
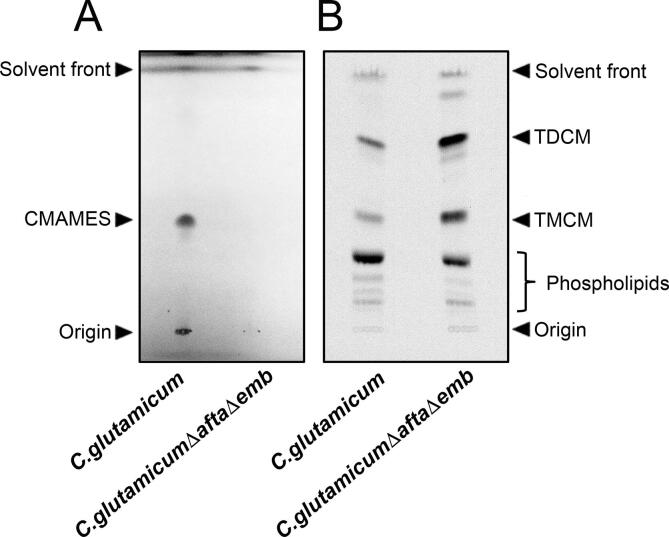
Analysis of cell wall bound (A) and cell wall associated (B) corynomycolic acids from *C. glutamicum* and *C. glutamicumΔaftAΔemb*. (A) Cell wall bound lipids were released from delipidated cells by addition of tetra-butylammonium hydroxide at 100 °C and methylated. An equivalent aliquot from each strain was subjected to TLC using silica gel plates (5735 silica gel 60F254, Merck), and developed in petroleum ether:acetone (95:5, v/v) to reveal CMAMEs. (B) Freely extractable lipids were released using chloroform:methanol:water (10:10:3; v/v/v), washed and subjected to TLC using silica gel plates (5735 silica gel 60F254, Merck). TLC plates were developed in chloroform:methanol:ammonium hydroxide (80:20:2, v/v/v) to separate [^14^C]-labelled trehalose dicorynomycolates (TDCM) and trehalose monocorynomycolates (TMCM).

Both *C. glutamicum* and *C. glutamicumΔaftaΔemb* strains were analysed for AG-esterified corynomycolic acids and cell wall associated lipids from an equivalent starting amount of biomass for each strain due to differences in growth rate. As expected, cell wall bound corynomycolic acids, analysed as CMAMES, were completely abolished in the *C. glutamicumΔaftaΔemb* mutant indicating a major defect in cell wall biosynthesis in comparison to the cell wall of *C. glutamicum* (Fig. 2A). Analysis of the cell wall associated lipids in the mutant highlighted an apparent increase in trehalose dicorynomycolates (TDCM) and trehalose monocorynomycolates (TMCM), the equivalent of mycobacterial TDMs and TMMs, respectively (Fig. 2B). This was verified quantitatively by labelling cultures with [^14^C]-acetate and loading equal amounts of radioactive extractable free lipids from *C. glutamicum* and *C. glutamicumΔaftaΔemb* (Fig. 2B). Densitometry based analysis of the *C. glutamicumΔaftaΔemb* mutant demonstrated a significant increase in TDCMs (275%) and TMCMs (141%), when compared to the wild type strain (Fig. 2B). These cell wall lipid phenotypes are consistent with the previous studies of the single *C. glutamicumΔaftA* and *C. glutamicumΔemb* mutants (Alderwick et al., 2006b, Alderwick et al., 2005). The continued synthesis of TDCM and TMCM suggests that by perturbing AG biosynthesis, potential mycolation sites have been removed and these mycolate biosynthetic precursors have accumulated.

### Structural characterisation of AG isolated from *C. glutamicum* and *C. glutamicumΔaftAΔemb*

**Fig. 3:**
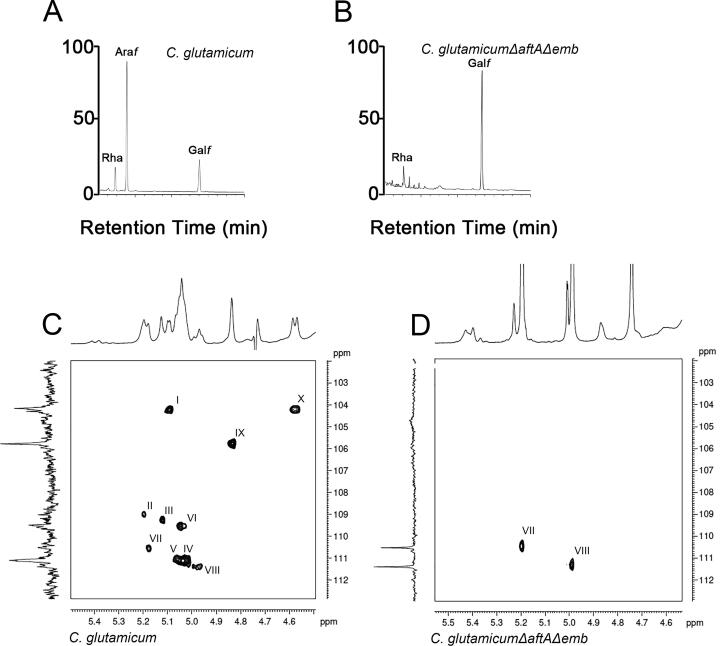
Structural characterisation of AG isolated from *C. glutamicum* and *C. glutamicumΔaftAΔemb*. Glycosyl compositional analysis of cell walls (mAGP) of *C. glutamicum* (A) and *C. glutamicumΔaftAΔemb* (B). The two-dimensional NMR spectra of AG purified from *C. glutamicum* (C) and *C. glutamicumΔaftA*Δ*emb* (D). ^1^H, ^13^C HSQC NMR spectra were acquired in D_2_O at 313 K. Expanded regions (δ ^1^H: 5.0–5.30, δ ^13^C: 101–111) are shown. *t*-β-Ara*f* (I), 2-α-Ara*f* → 3 (II), 2-α-Ara*f* → 5 (III), 5-α-Ara*f* (IV), 3,5-α-Ara*f* (V), 2,5-α-Ara*f* (VI), 5-β-Gal*f* (VII), 6-β-Gal*f* (VIII), *t*-α-Rha*p* (IX and X) and 3-α-Ara*f* (XI) are highlighted.

In order to determine the major cell wall sugar composition of the *C. glutamicumΔaftAΔemb* double deletion mutant, highly purified mAGP cell wall material was isolated from both *C. glutamicum* and *C. glutamicum*Δ*aftA*Δ*emb* (Alderwick et al., 2005, Seidel et al., 2007). The mAGP was then chemically derivatised to alditol acetates and subsequently analysed by gas chromatography (GC) (Alderwick et al., 2005, Seidel et al., 2007). GC analysis of alditol acetates prepared from *C. glutamicum* mAGP demonstrated the presence of rhamnose, arabinose and galactose with an approximate arabinose to galactose ratio of 2.8:1 (Fig. 3A) (Alderwick et al., 2006b, Alderwick et al., 2005, Alderwick et al., 2018, Seidel et al., 2007). In contrast, alditol acetates derived from *C. glutamicum*Δ*aftA*Δ*emb* mAGP revealed a total absence of arabinose (Fig. 3B).

Analysis of the base-solubilised AG from *C. glutamicum* and *C. glutamicum*Δ*aftA*Δ*emb* was performed using NMR spectroscopy. The ^1^H NMR spectrum of wild type *C. glutamicum*-AG (Fig. 3C) was highly complex when compared to the anomeric region of *C. glutamicum*Δ*aftA*Δ*emb* ‘AG’ (Fig. 3D). The obtained spectral data was compared previously published spectra (Birch et al., 2010, Jankute et al., 2017, Lee et al., 2005, Liu et al., 2011, Mahrous et al., 2008), from which we were able to fully assign each of the resonances I-X (Fig. 3C-D). The ^13^C resonance I at δ 104.2 ppm correlates to an anomeric proton at δ 5.1 ppm, and was assigned as the t-β-Ara*f* → 5 linkage. The resonances II and III at δ 108.9 ppm and δ 109.2 ppm, correlated to protons at δ 5.2 ppm and δ 5.12 ppm, and were assigned as 2-α-Ara*f* → 3 and 2-α-Ara*f* → 5 linkages, respectively (Fig. 3C). Peak IV (δ 111.1 ppm and δ 5.06 ppm) was assigned to 5-α-Ara*f* linkage and was observed to overlap with a peak, which was assigned to a 3,5-α-Ara*f* (δ 111.1 ppm −δ 5.02 ppm) linkage for wild-type *C. glutamicum*-AG. The ^13^C resonance VI at δ 109.4 ppm, which correlated to a proton at δ 5.05 ppm was designated as the 2,5-α-Ara*f* linkage (Fig. 3D). The well separated peaks VII and VIII for 5-β-Gal*f* (δ 110.6 ppm, δ 5.18 ppm) and 6-β-Gal*f* (δ 111.4 ppm, δ 4.98 ppm) were visible in both wild type *C. glutamicum* and *C. glutamicum*Δ*aftA*Δ*emb*-‘AG’ spectra (Fig. 3C-D). Overall, ^1^H, ^13^C HSQC 2D-NMR analysis indicates that the ‘AG’ of the double *C. glutamicum*Δ*aftA*Δ*emb* mutant is lacking arabinan and only possesses unaltered cell wall galactan, which confirms the earlier glycosyl compositional analysis.

### *In vitro* arabinofuranosyltransferase activity with membrane extracts from *C. glutamicum* and mutant strains

**Fig. 4:**
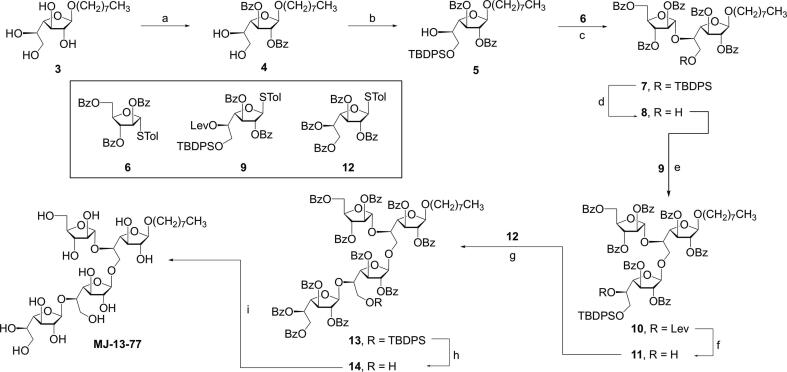
Synthesis of MJ-13-77. a) 2,2-DMP, acetone, cat. *p*-TSA; then BzCl, pyridine; then AcOH–H_2_O–THF (3:1.5:1.5), 61% three steps; b) TBDPSCl, CH_2_Cl_2_, pyridine, 96%; c) NIS, AgOTf, CH_2_Cl_2_; d) HF·pyridine, THF–pyridine, 76% (two steps); e) NIS, AgOTf, CH_2_Cl_2_, 90%; f) H_2_NNH_2_·AcOH, 91%; g) NIS, AgOTf, CH_2_Cl_2_, 89%; h) HF·pyridine, THF, pyridine, 91%; i) NaOCH_3_, CH_3_OH, CH_2_Cl_2_, quant.

**Fig. 5:**
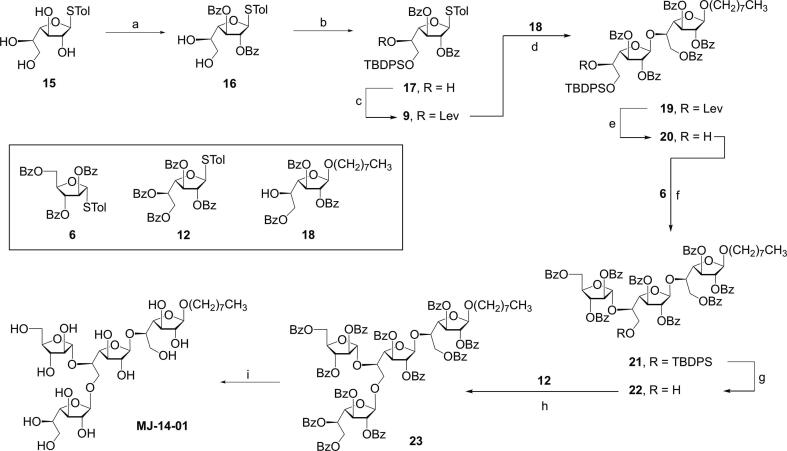
Synthesis of MJ-14-01. a) 2,2-DMP, acetone, cat. *p*-TSA; then BzCl, pyridine; then AcOH–H_2_O–THF (3:1.5:1.5), 56% three steps; b) TBDPSCl, CH_2_Cl_2_, pyridine; c) Levulinic acid, DCC, DMAP, 93% (two steps); d) NIS, AgOTf, CH_2_Cl_2_; e) H_2_NNH_2_·AcOH, 84% (two steps); f) NIS, AgOTf, CH_2_Cl_2_, 93%; g) HF·pyridine, THF,pyridine, 88%; h) NIS, AgOTf, CH_2_Cl_2_, 90%; i) NaOCH_3_, CH_3_OH, CH_2_Cl_2_, quant.

**Fig. 6:**
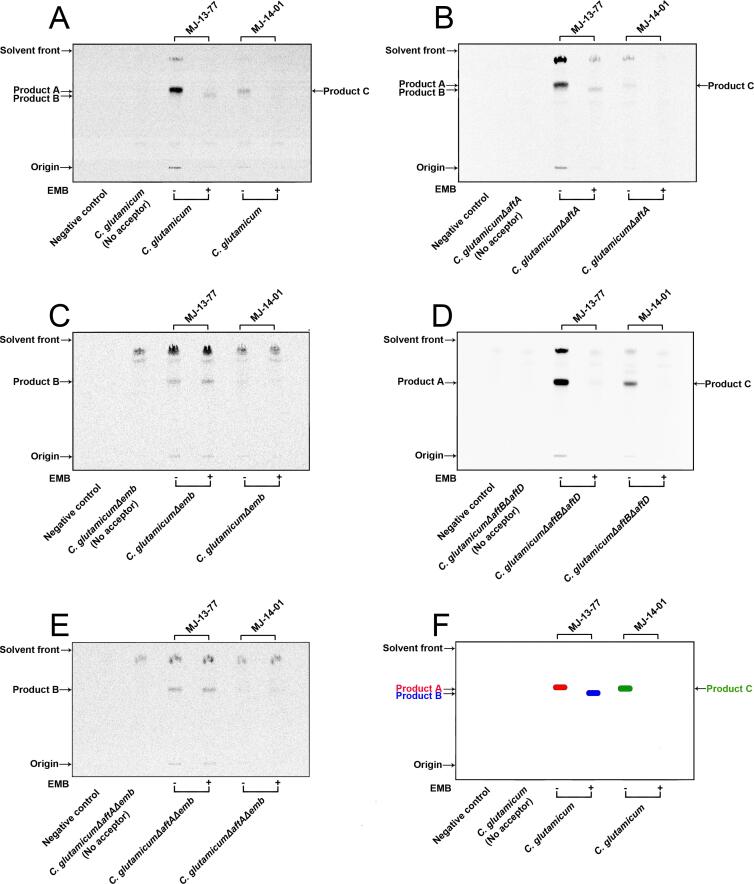
Arabinofuranosyltransferase activity assay utilising MJ-13-77 and MJ-14-01 neoglycolipid acceptors, p[^14^C]Rpp and membranes prepared from *C. glutamicum* (A), *C. glutamicumΔaftA* (B), *C. glutamicumΔemb* (C), *C. glutamicumΔaftBΔaftD* (D), *C. glutamicumΔaftAΔemb* (E) and representative diagram of products (F). Arabinofuranosyltransferase activity was determined using synthetic acceptors in a cell-free assay with and without ethambutol (EMB). The products of the assay were processed and subjected to TLC using silica gel plates (5735 silica gel 60F254, Merck) in isopropanol:acetic acid:water (8:1:1, v/v/v) with the reaction products visualised by either autoradiography using Kodak BioMax MR films or phosphorimaging using Molecular Imager FX (Bio-Rad). Negative controls represent reactions performed without acceptors and membranes preparations.

Our initial attempts to develop an *in vitro* assay using purified recombinant Emb from *C. glutamicum* have thus far proved unsuccessful due to the formation of inclusion bodies. As an alternative, we analysed Ara*f*T activity in a cell-free assay in the presence of exogenous neoglycolipid acceptors, β-D-Gal*f*-(1 → 5)-β-D-Gal*f*-(1 → 6)[α-D-Ara*f*-(1 → 5)]-β-D-Gal*f*-*O*-(CH2)7CH3 [**MJ-13**-**77**, Fig. 4] and β-D-Gal*f*-(1 → 6)-[α-D-Ara*f*-(1 → 5)]-β-D-Gal*f*-(1 → 5)-β-D-Gal*f*-*O*-(CH2)7CH3 [**MJ-14**-**01**, Fig. 5], p[^14^C]Rpp and membrane preparations from *C. glutamicum*, *C. glutamicumΔaftA*, *C. glutamicumΔemb*, *C. glutamicumΔaftBΔaftD* and *C. glutamicumΔaftAΔemb*. The cell-free assay format was based on previously established Ara*f*T assays (Birch et al., 2008, Seidel et al., 2007) and was designed to directly examine whether the Emb protein from *C. glutamicum* may transfer a second Ara*f* residue in an α(1 → 5) fashion to the already Ara*f* “primed” galactan chain. Assays were conducted both in the presence and absence of EMB in order to inhibit the single *C. glutamicum* Emb protein. The [^14^C]-labelled products were extracted using organic solvents, separated by TLC and detected by autoradiography. Although, the efficiency of **MJ-14**-**01** and **MJ-13**-**77** were lower than our previously described Ara*f*T acceptors (Birch et al., 2008, Seidel et al., 2007), three bands were observed migrating at R_f_ of 0.66, R_f_ of 0.61 and R_f_ of 0.64 and were labelled as Products A, B and C, respectively (see Fig. 6F). Assays conducted using wild type *C. glutamicum* membranes and acceptor **MJ-13**-**77** produced a TLC autoradiogram with a product profile consisting of two bands annotated as Product A (major) and Product B (minor), respectively (Fig. 6A). For acceptor **MJ-14**-**01**, a single band was observed, which was annotated as Product C (Fig. 6A). Addition of EMB to the assay mixture resulted in inhibition of Products A and C, suggesting that the enzyme adding a Ara*f* residue onto these acceptors is EMB sensitive, and therefore most likely is added by the single Emb Ara*f*T in *C. glutamicum*. These experiments also allowed the minor Product B to be visualised more clearly. A similar experiment was repeated using *C. glutamicumΔaftA* membranes and **MJ-13**-**77** and **MJ-14**-**01**, and a similar profile to that of wild-type *C. glutamicum* was observed indicating that deletion of Cg-AftA does not participate in the generation of Products A-C (Fig. 6B). Experiments utilising membranes devoid of Cg-Emb activity from *C. glutamicumΔemb* and **MJ-13**-**77** and **MJ-14**-**01** were performed and both Products A and Product C were not generated (Fig. 6C), strongly indicating that the deleted enzyme Cg-Emb is indeed responsible for the generation of these two [^14^C]-labelled products observed by TLC-autoradiography. Interestingly, the *C. glutamicumΔemb* strain was still able to produce Product B utilising **MJ-13**-**77**. Addition of EMB to the reaction mixture did not inhibit the synthesis of Product B, thus implying that Ara*f*T adding the Ara*f* residue to the MJ-13-77 is EMB insensitive. The *in vitro* experiment was repeated using *C. glutamicumΔaftBΔaftD* membranes and the product profile closely resembled that of the assay carried out using wild type *C. glutamicum* with and without EMB (Fig. 6D)*.* The data thus suggests that neither AftB with β (1 → 2) (Seidel et al., 2007) or AftD with α(1 → 5) Ara*f*T activities (Alderwick et al., 2018a), respectively, contribute to the Ara*f* addition to either **MJ-13**-**77** or **MJ-14**-**01**. The data collectively suggests that the formation of Product B is generated by AftC (Birch et al., 2008), which was shown to possess an α(1 → 3) Ara*f*T activity and is EMB insensitive. Finally, *in vitro* Ara*f*T assays were performed using *C. glutamicumΔaftAΔemb* membranes and the acceptors leading to formation of only Product B (Fig. 6E). The combination of visible bands on the TLC is similar to the one produced utilising *C. glutamicumΔemb* membranes, indicating that formation of both Products A and C is due to Emb enzymatic activity. Overall, the TLC product profiles obtained show that Products A and C were formed as a result of Emb Ara*f*T activity transferring [^14^C]-Ara*f* to the **MJ-13**-**77** and **MJ-14**-**01** acceptors, respectively.

### Mass spectrometry analysis of *in vitro* generated products A-C

**Fig. 7:**
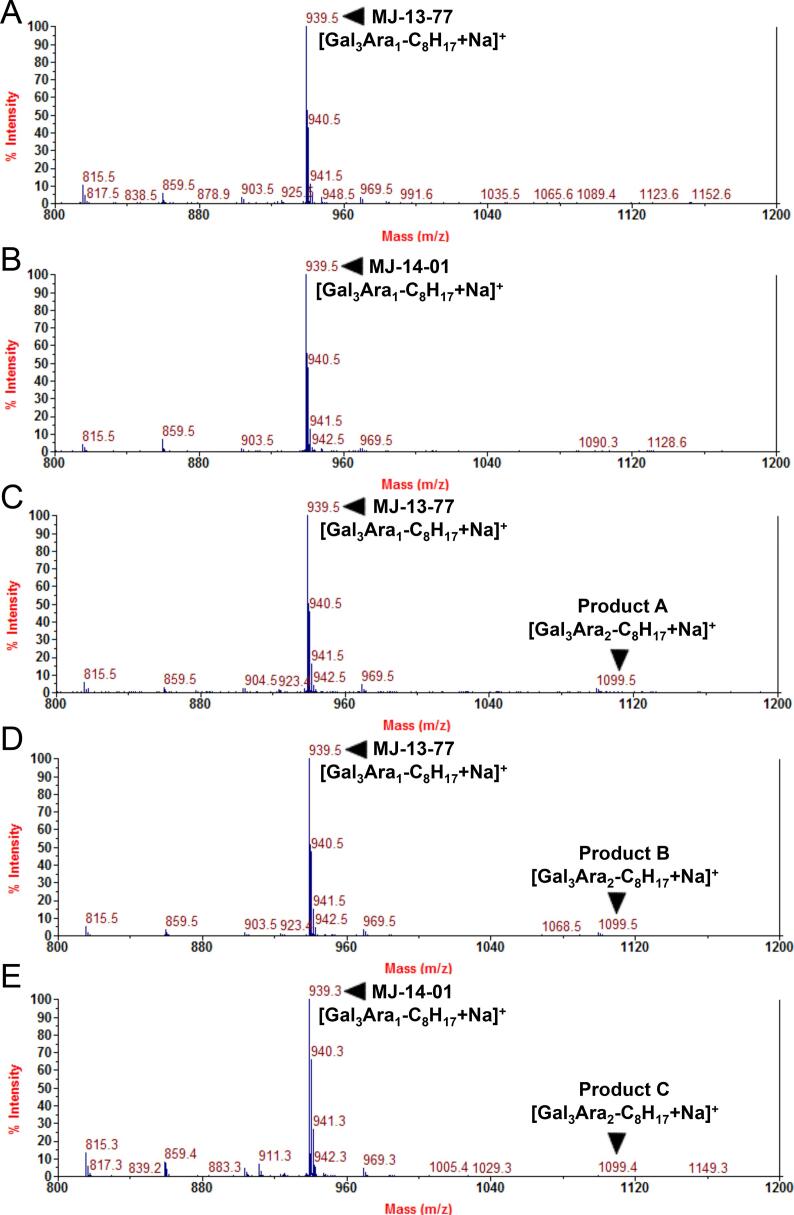
MALDI-TOF MS analysis of acceptors and enzymatic products. Both β-D-Gal*f*-(1 → 5)-β-D-Gal*f*-(1 → 6)[α-D-Ara*f*-(1 → 5)]-β-D-Gal*f*-*O*-(CH2)7CH3 (MJ-13-77) (A) and β-D-Gal*f*-(1 → 6)-[α-D-Ara*f*-(1 → 5)]-β-D-Gal*f*-(1 → 5)-β-D-Gal*f*-*O*-(CH2)7CH3 (MJ-14-01) (B) were dried, per-*O*-methylated and subjected to MALDI-TOF MS. (C–E) Products formed utilising Gal_3_Ara_1_-C_8_H_17_ synthetic acceptors and wild-type *C. glutamicum* membranes were extracted from the preparative TLC and samples prepared for MALDI-TOF MS.

Products A, B and C, synthesised using synthetic acceptors **MJ-13**-**77** and **MJ-14**-**01**, non-radiolabelled pRpp and membranes from wild-type *C. glutamicum*, and in some cases EMB treatment were excised from preparative TLC plates, per-*O*-methylated and subjected to MALDI-TOF MS analysis. To rule out co-chromatography of the starting material with the products even after separation by TLC, due to similar R_f_ values, both **MJ-13**-**77** and **MJ-14**-**01**, were also analysed and served as a marker for any remaining unreacted acceptor (Fig. 7A, B). All three samples containing Products A, B and C have provided similar molecular ion profiles (Fig. 7C–E). Molecular ions of *m/z* 1099 was observed and corresponded to a pentasaccharide product(s) [Gal_3_Ara_2_-C_8_H_17_ + Na]^+^ (Fig. 7C–E) containing a newly-added Ara*f*-(1 → ?) residue. Ions for unreacted **MJ-13**-**77** and **MJ-14**-**01** of *m/z* 939 [Gal_3_Ara_1_-C_8_H_17_ + Na]^+^ were also detected (Fig. 7C–E). In addition, all samples contained molecular ions of *m/z* 969, suggesting a possible per-*O*-methylation artefact. Overall, the MALDI-TOF MS analysis confirmed the addition of only a single Ara*f* residue to **MJ-13**-**77** and **MJ-14**-**01**, resulting in three pentasaccharide Products A, B and C with composition of Gal_3_Ara_2_-C_8_H_17_.

**Fig. 8:**
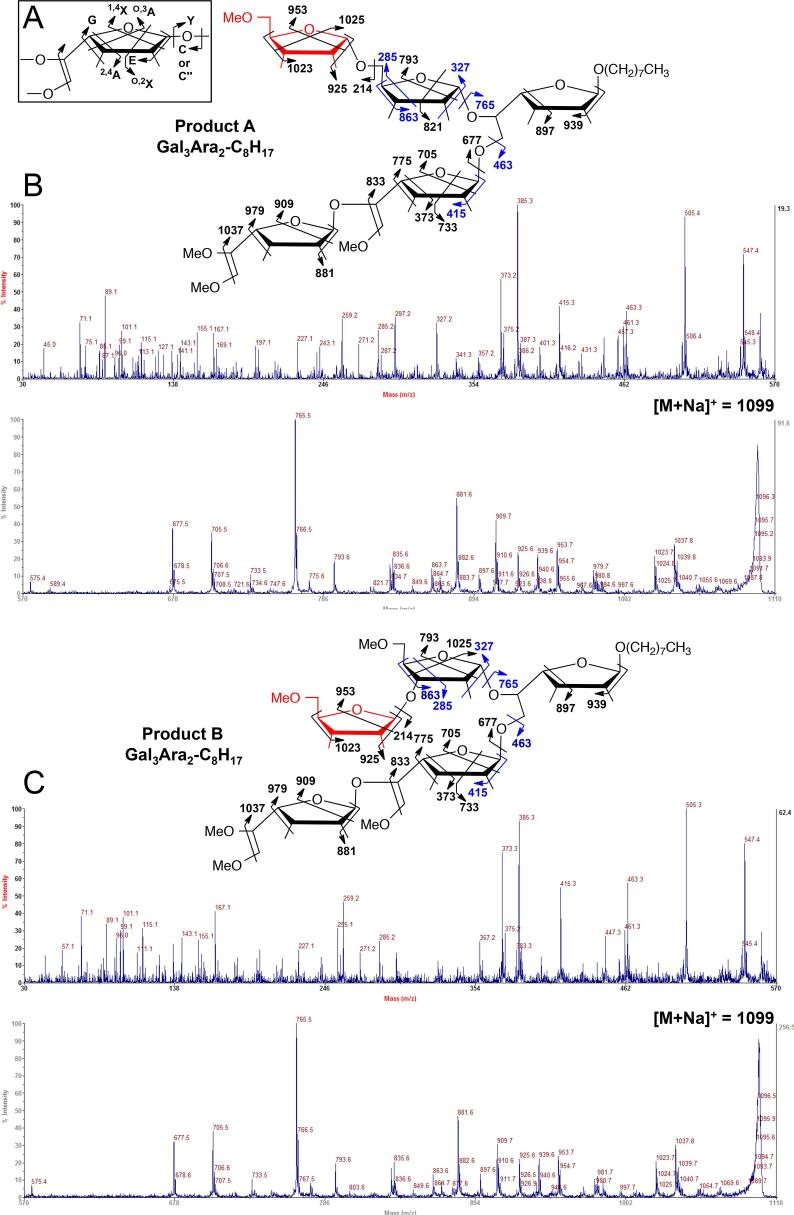
MALDI-TOF MS/MS analysis (A–C) of per-*O*-methylated Product A (B) and Product B (C). The added Ara*f* residue is depicted in red, whereas cleavages and linkages that are diagnostic for determining the structure are depicted in blue. (For interpretation of the references to colour in this figure legend, the reader is referred to the web version of this article.)

**Fig. 9:**
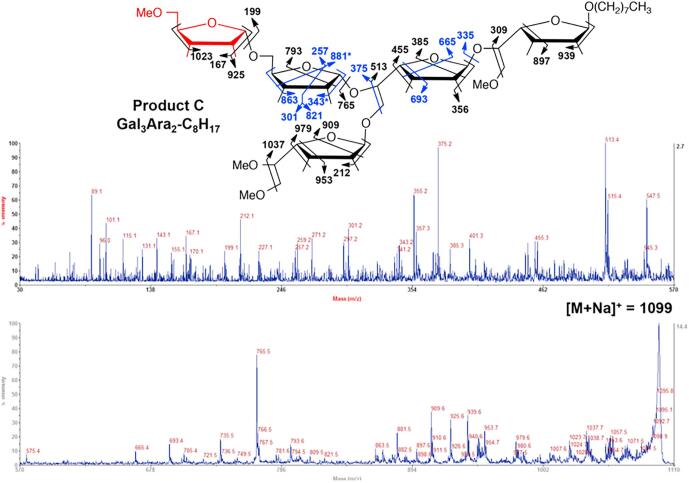
MALDI-TOF MS/MS analysis of per-*O*-methylated Product C. The added Ara*f* residue is depicted in red, whereas cleavages and linkages that are diagnostic for determining the structure are depicted in blue. (For interpretation of the references to colour in this figure legend, the reader is referred to the web version of this article.)

In order to define the branching pattern of the generated oligomers, the respective ions were subjected to MALDI-TOF MS/MS analysis. The fragmentation patterns of various synthetic acceptors and arabinan oligomers have been validated previously (Lee et al., 2006, Shi et al., 2008, Zhang et al., 2007). The original Gal_3_Ara_1_-C_8_H_17_ acceptors provided a series of ions: ^O,3^A, ^2,4^A, C, E, G, ^O,2^X and ^1,4^X (Fig. 8A), consistent with expected linkages and cleavage ions of neoglycolipid acceptors. Both Product A and B provided similar MS/MS spectra with common C and E ions at *m/z* 463 and 415, respectively, indicating that in both cases an Ara*f* residue has been added onto the existing Ara*f* residue of the **MJ-13**-**77** acceptor (Fig. 8B, C). Lack of arabinosylation at the C-2 of the Ara*f* residue of **MJ-13**-**77** is supported by the absence of ions ^2,4^A and ^O,2^X of *m/z* 125 and 981, respectively. The Y ion of *m/z* 765, coupled with ions at *m/z* 285, 327 and 863, locates the new Ara*f* residue at either C-3 or C-5 of the existing Ara*f* unit of the **MJ-13**-**77** acceptor in Products A and Product B (Fig. 8B, C). Since, earlier studies have shown that α(1 → 5) Ara*f*T activity is EMB sensitive, this would suggest that Product A is probably formed by the addition of a new Ara*f* residue at C-5, while Product B at C-3, as it is ethambutol insensitive (Fig. 6). In contrast, Product C (Fig. 9) is formed by Emb activity yielded the Y, ^O,3^A and ^2,4^A ions at *m/z* 335, 665 and 693, respectively, indicated that the newly attached Ara*f* residue is linked to the Ara*f* residue of the **MJ-14**-**01** acceptor. The ion of *m/z* 375 clearly places the Ara*f* residue to the α(1 → 5) linked Ara*f* of the acceptor, which mimics the Ara*f*-“primed” galactan chain. Unfortunately, the diagnostic ^O,3^A, ^O,2^X, E and G ions with *m/z* of 257, 301, 343 and 863, respectively, that would define the Ara*f* residue specifically to C-5 were not unique in this spectra (Fig. 9). However, it is most likely to be added at C-5 of the **MJ-14**-**01** acceptor due to its observed sensitivity to EMB.

Considering the TLC, MS analysis and EMB-sensitivity data altogether, it is possible to conclude that the pentasaccharide Product A is formed by the single Emb protein, which acts as an α(1 → 5) Ara*f*T and transfers the Ara*f* residue to the C-5 of the **MJ-13**-**77**. In contrast, the generation of pentasaccharide Product B was observed even in the presence of EMB, indicating that the Ara*f*T is EMB insensitive, and mostly likely an α(1 → 3) Ara*f*T (AftC), as other AftTs have been sequentially ruled out through either genetic, MS data or EMB sensitivity data (AftA, AftB and AftD) (Fig. 6A–E). Likewise, Product C results from the addition of an Ara*f* unit to **the MJ-14**-**01** attached at position C-5, which was also EMB sensitive (Fig. 9). Our results establish for the first time that Emb from *C. glutamicum* acts as α(1 → 5) Ara*f*T and results in the polymerisation of a suitably-primed Ara*f*-galactan chain.

## Discussion

The mAGP complex is a key cell wall component of the *Corynebacterianeae*, which is essential for the growth and viability of *M. tuberculosis*. This highly complex structure is rich in lipids and sugars that act as a low permeability barrier and contributes to resistance to common antibiotics. It is therefore not surprising that potent anti-TB drugs target the biosynthesis of mAGP, including EMB and isoniazid, both of which inhibit the biosynthesis of AG and mycolic acids, respectively. Nevertheless, cases of MDR and XDR-TB pose a serious challenge to treatments offered by chemotherapy, increasing the need to discover novel drug targets and the development of new therapeutic agents against *M. tuberculosis* infections. Our current knowledge of Ara*f*Ts that synthesise the arabinan domains of both AG and LAM remains somewhat incomplete. Recently, a novel mycobacterial Ara*f*T activity was described for LAM biosynthesis, but the specific enzyme remains to be identified and characterised (Angala et al., 2016). In addition, the catalytic mechanisms and complex protein interactions of how different Ara*f*Ts synthesise the bulk of the arabinan, containing α(1 → 5), α(1 → 3) and β(1 → 2) glycosidic linkages in mycobacteria and corynebacteria remain unclear. Structural and functional information on these enzymes would aid the further exploitation of Ara*f*Ts as potential drug targets to inhibit the crucial mAGP complex in *M. tuberculosis*.

Previously described Ara*f*Ts include the Emb proteins (Alderwick et al., 2005, Escuyer et al., 2001), AftA (Alderwick et al., 2006b), AftB (Jankute et al., 2017, Seidel et al., 2007), AftC (Birch et al., 2008) and AftD (Alderwick et al., 2018, Skovierová et al., 2009), which all share certain functional relationships, but also possess distinct Ara*f*Ts with their own individual characteristics. A classic example is the sensitivity of Emb proteins from *Mycobacterium* species and Emb from *C. glutamicum* towards EMB, and the insensitivity of other Ara*f*Ts including AftA, AftB and AftC towards the same drug. The number of Ara*f*Ts involved in the assembly of the arabinan domain in AG remains a matter of speculation. However, the current structure of mycobacterial AG suggests at least six different Ara*f*Ts that are involved in its biosynthesis. Since, Emb proteins are the targets of EMB, a number of studies here attempted to characterise this role in AG biosynthesis. Individual EmbA and EmbB deletion mutants in *M. smegmatis* possessed reduced levels of the disaccharide β-D-Ara*f*-(1 → 2)-α-D-Ara*f* in AG resulting in a terminal non-reducing Ara_4_ motif instead of the usual Ara_6_ motif (McNeil et al., 1991). It was concluded that EmbA and EmbB are responsible for the 3,5 branching in AG and the synthesis of a distinct disaccharide β-D-Ara*f*-(1 → 2)-α-D-Ara*f* (Escuyer et al., 2001). However, direct evidence for site of action i.e. the Ara_6_ domain *via* 3,5 branching in AG or early stages of arabinan biosynthesis has been lacking. It is intriguing also that one can delete successfully Cg-Emb and obtain a slow-growing phenotype with a highly truncated AG-glycan with single arabinose residues attached to the galactan core, whereas *M. tuberculosis* produces a more matured AG-glycan in both EmbA and EmbB mutants, suggesting possibly that the Emb proteins from *C. glutamicum* and *M. tuberculosis* may in fact functional differ in their arabinosyltransferase activities.

In this study, we have characterised a *C. glutamicumΔaftAΔemb* double deletion mutant. Analysis of its cell wall revealed an AG containing only the galactan backbone of AG with no arabinose residues (Fig. 3). In addition, the mutant led to an increase in non-covalently linked TDCMs and TMCMs and a lack of covalently bound cell wall mycolates indicating the loss of mycolylation sites in AG (Fig. 2A, B). By employing a cell-free assay using *C. glutamicum*, *C. glutamicumΔaftA*, *C. glutamicumΔemb*, *C. glutamicumΔaftBΔaftD*, *C. glutamicumΔaftAΔemb* strains, and neoglycolipid acceptors that resemble the Ara*f-*primed galactan of AG, and subsequent analysis of the products formed, we have shown that the transfer of a Ara*f* residue from DPA to the Ara*f*-primed galactan in *C. glutamicum* is catalysed by the EMB sensitive Emb Ara*f*T with α(1 → 5) activity.

The discovery of Emb from *C. glutamicum* as an α(1 → 5) Ara*f*T has shed further light on the key Ara*f*Ts involved in the synthesis of AG, which may contribute to a more detailed understanding of pathogenicity and persistence of *M. tuberculosis*. The challenge for the future will be to define the precise role of further Ara*f*Ts in *Mycobacterium* species, scrutinise their catalytic mechanisms and formation of protein complexes in order to synthesise AG.

## Declaration of Competing Interest

The authors declare that they have no known competing financial interests or personal relationships that could have appeared to influence the work reported in this paper.
